# Discovery of novel fungal species and pathogens on bat carcasses in a cave in Yunnan Province, China

**DOI:** 10.1080/22221751.2020.1785333

**Published:** 2020-07-09

**Authors:** Samantha Chandranath Karunarathna, Yang Dong, Seigi Karasaki, Saowaluck Tibpromma, Kevin David Hyde, Saisamorn Lumyong, Jianchu Xu, Jun Sheng, Peter Edward Mortimer

**Affiliations:** a CAS Key Laboratory for Plant Diversity and Biogeography of East Asia, Kunming Institute of Botany, Chinese Academy of Science, Kunming, Yunnan, People’s Republic of China; b State Key Laboratory for Conservation and Utilization of Bio-Resources in Yunnan, Yunnan Agricultural University, Kunming, People’s Republic of China; c Yunnan Research Institute for Local Plateau Agriculture and Industry, Kunming, People’s Republic of China; d Key Laboratory for Agro-biodiversity and Pest Control of Ministry of Education, Yunnan Agricultural University, Kunming, People’s Republic of China; e Energy and Resources Group, University of California, Berkeley, CA, USA; f World Agroforestry Centre, Kunming, Yunnan, People’s Republic of China; g Centre for Mountain Futures, Kunming Institute of Botany, Kunming, Yunnan, People’s Republic of China; h Center of Excellence in Fungal Research, Mae Fah Luang University, Chinag Rai, Thailand; i Department of Biology, Faculty of Science, Chiang Mai University, Chiang Mai, Thailand; j Center of Excellence in Microbial Diversity and Sustainable Utilization, Faculty of Science, Chiang Mai University, Chiang Mai, Thailand; k Academy of Science, The Royal Society of Thailand, Bangkok, Thailand

**Keywords:** 4 new taxa, *Fusarium incarnatum*, *Mucor hiemalis*, *Mortierella rhinolophicola*, *Mortierella yunnanensis*, *Mortierella multispora*, *Neocosmospora pallidimors*, *Trichoderma harzianum*

## Abstract

Virulent infectious fungal diseases, in natural and managed landscapes, are increasing. Fungal diseases in humans, animals and plants have caused die-off and extinction events and have become a threat to food security. A caving expedition in Yunnan Province, China, revealed two bat carcasses covered in fungal mycelia. Eleven fungal isolates were obtained from these bat carcasses, and morphological observations and multigene phylogenetic analyses revealed they were *Fusarium incarnatum, Mucor hiemalis* and *Trichoderma harzianum* and four new species, *Mortierella rhinolophicola, M. multispora, M. yunnanensis* and *Neocosmospora pallidimors*. One of the more alarming findings is that a number of infections related to *Neocosmospora*, previously associated with human and animal mycotoxicoses, are reported to be increasing, and here we present a new species from this genus, isolated from dead bats. Due to the ecosystem services provided by bats, and the close relationship between bats and humans, future research should focus on the impacts and significance of N. pallidimors to human and animal health, examining its pathogenicity and secondary metabolites. Taxonomic descriptions, color images of the habitat, *in situ* samples, microstructures and cultures are presented. SEM photographs of microstructures and phylogenetic trees showing the placement of new and known species are also provided.

## Introduction

Cave ecosystems are unique windows into microbial and fungal life in the subsurface of the Earth. Living in oligotrophic environments with limited light and nutrients, organisms endemic to cave ecosystems exhibit photoautotrophic and photoperiodic adaptations [1]. Research documented a high level of microbial and bacterial diversity in hypogean systems. Depending on physical, ecological, and environmental characteristics, cave systems host hyper-localized speciation and ecologies [2]. Given our limited knowledge on hypogean biological diversity both at regional and local scales, cave systems – including, but not limited to, those winding through limestone karsts – are a reservoir of new and undiscovered species.

These caves, and the animals inhabiting them, are also a source of human pathogens and diseases [3]. Severe acute respiratory syndrome coronavirus (SARS-CoV) and the current worldwide coronavirus (Covid-19) epidemic are just two examples that have had far-reaching impacts on society. Although slightly outdated, the review articles by Igreja [4] and Jurado et al. [3] document the different diseases and pathogens found in cave systems. Of subterranean interactions, pathogenic relationships between cave fauna and fungi warrant particular attention. In recent years, fungal and fungal-like diseases have caused die-off and extinctions events of host species at unprecedented scales [5]. For cave fauna, the devastating effect of *Pseudogymoascus destructans* (known as white-nose disease) is the most notorious example: in 2006, white-nose disease rapidly spread across North America, causing mass mortality and regional population collapse in the little brown myotis (*Myotis lucifugus*), a hibernacula [6]. As European bats are known to asymptomatically carry *P. destructans*, it was hypothesized that *P. destructans* was introduced to susceptible North American bats from the Old World, probably via human transport [7]. Furthermore, *Histoplasma capsulatum*, a human pathogenic and dimorphic fungus known to cause the disease Histoplasmosis in humans, is found in temperate areas throughout the world [8]. This fungus is found in soil and guano in caves and is carried by bats, although bats are usually asymptomatic. Current epidemics and past research suggest that cave ecosystems and bats pose ideal environments/hosts for the emergence of new pathogens and thus warrant further study.

The logistic difficulty of finding and accessing hypogean habitats has left them relatively undiscovered and unexplored [9]. Fungi are known to fill important ecological niches in cave systems, but there is scant scientific documentation detailing the relationship between the biological role(s) of cave microbes, fungi, flora, and fauna. The earliest cave fungi description was published by Humboldt in 1794 (as described in Dobat) [10]. By 2017, more than 1150 species of fungi, belonging to 550 genera, have been found in mines and caves worldwide [11,12]. The obligate troglobitic taxa, such as *Acaulium caviariforme*, *Aspergillus baeticus*, and *A. thesauricus* have been reported from mines and caves [11]. Interestingly, the majority of the recently discovered fungi from caves have also been discovered from other habitats [11–13]. Based on these findings, Zhang et al. [12] mentioned that cave fungi likely originated from non-cave habitats.

Little is known about the ecological origins and roles of hypogean fungi, such as where they originated, how they evolved, or if their evolutionary adaptations are localized. We fill a knowledge gap relating to the study of hypogean fungi with our presentation of new and known fungal species found on bat carcasses in a cave system in Yunnan Province, China. Furthermore, we provide detailed morphological and molecular descriptions of the fungal species isolated from the bat carcasses.

## Materials and methods

### Ethics statement

Bat carcasses were collected in a cave outside of Kunming City, Yunnan Province, China. The collection of material was performed under the following project codes: 41761144055, 41771063 and 2018PC0006, managed through the Kunming Institute of Botany, Chinese Academy of Sciences.

### Sample collection, specimen examination and isolation

**Figure 1.**
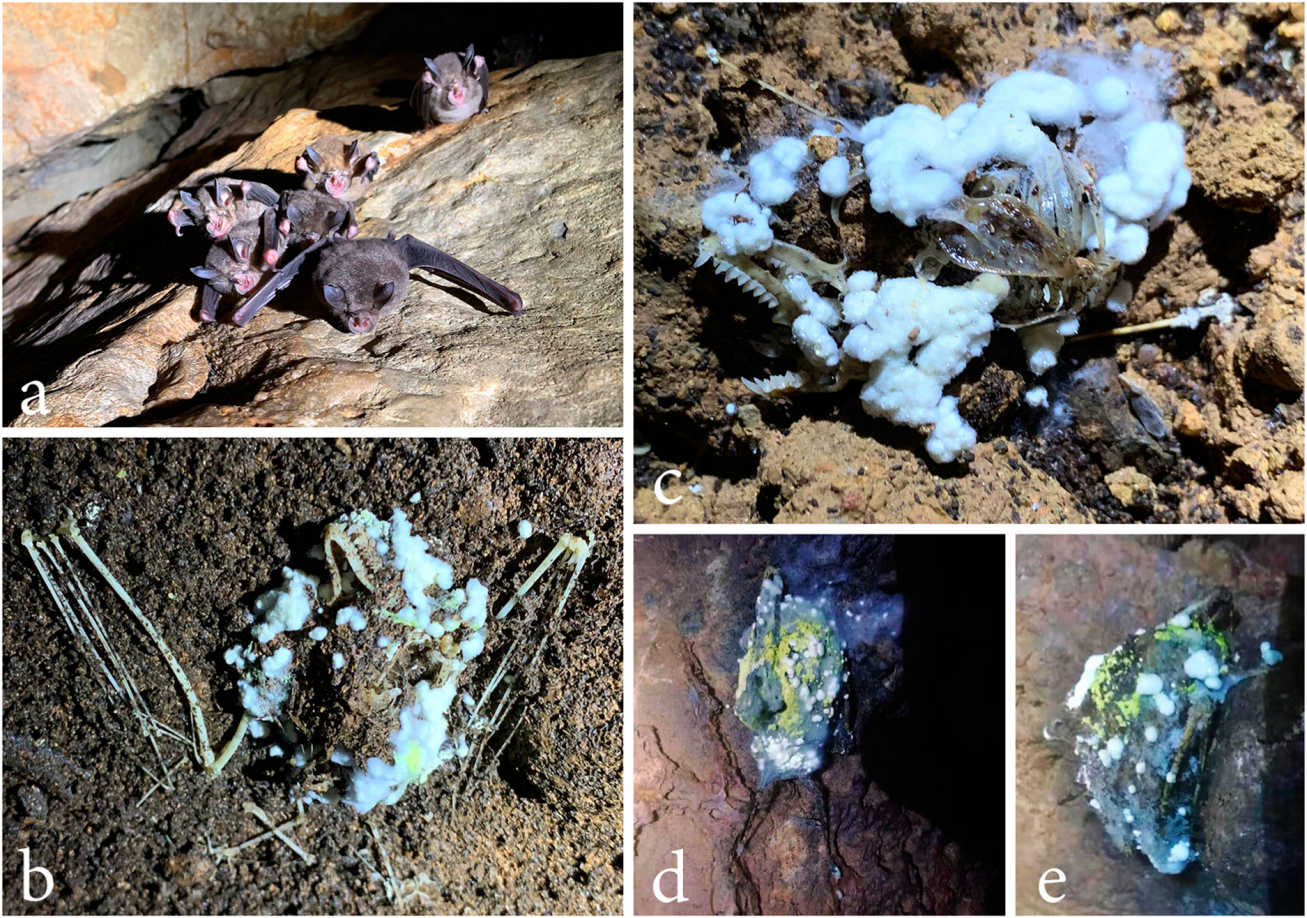
*Rhinolophus affinis* (horseshoe bats), and associated carcasses found in a cave outside Kunming City, Yunnan Province, China. a live bats roosting in the cave. b–e bat carcasses with white, green and yellow fungi mycelia.

**Figure 2.**
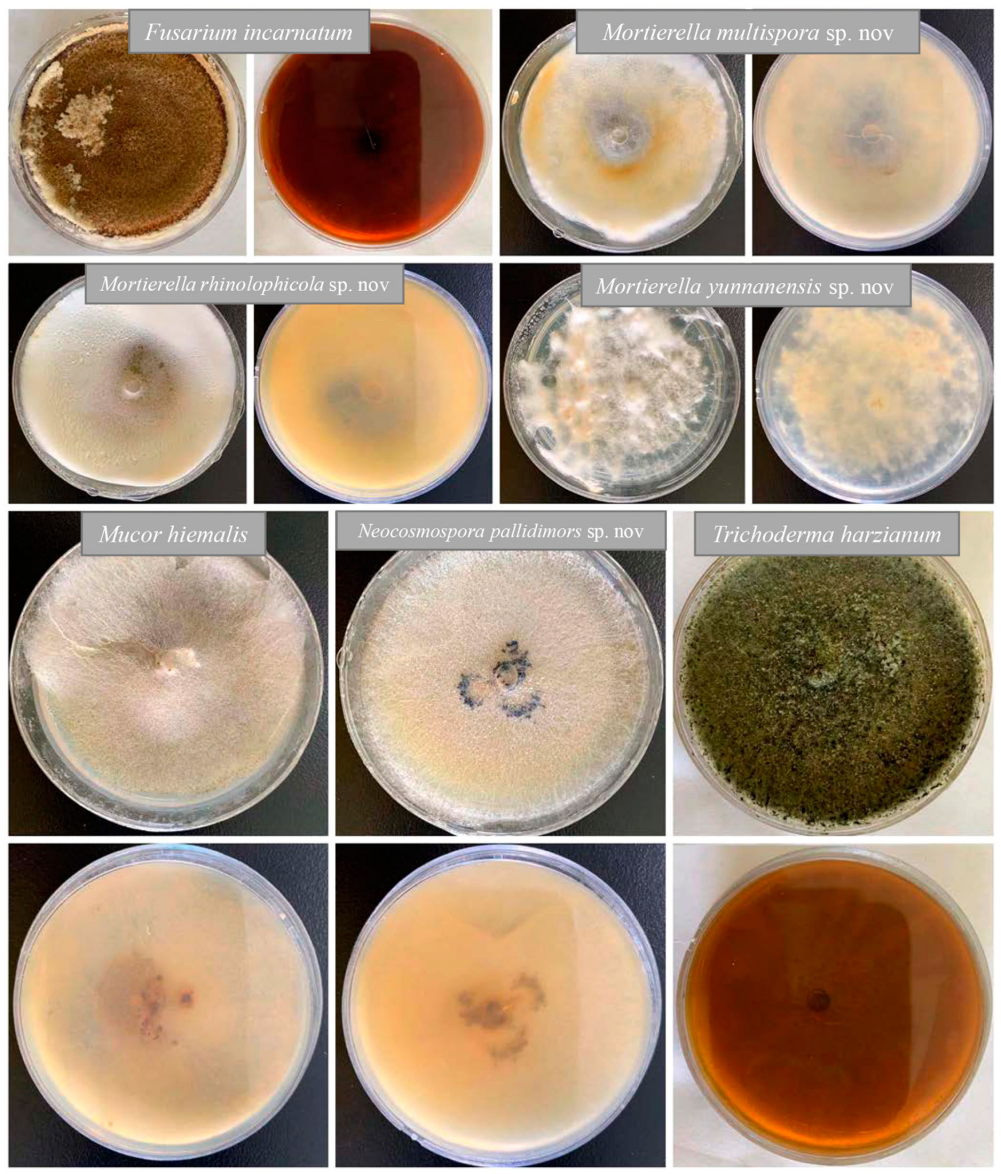
Cultures from this study are shown growing on PDA at 28°C after 4 months.

Two dead bats (*Rhinolophus affinis*) (Figure 1) covered with fungi were found on 5th August 2019, in a cave outside of Kunming City, Yunnan Province, China. Temperature, humidity, and carbon dioxide levels were recorded in caverns where the bat carcasses were found using a portable climate station (Xintest, HT-2000). Three climate readings were taken within the cave, and readings were recorded five minutes after the respective data values had stabilised. The fungi growing on the bat carcasses were isolated in Potato Dextrose Agar (PDA) using aseptic techniques. The cultures were placed at 28°C in an incubator. Observation of fungal morphological structures followed Wagner et al. [14], Sandoval-denis et al. [15], and Tibpromma et al. [16]. The pure cultures (Figure 2, Supplementary Table 1) were deposited at the Kunming Institute of Botany Culture Collection (KMUCC), while the herbaria of new species were deposited at the Key Laboratory of Industrial Microbiology and Fermentation Technology of Yunnan (YMF), Kunming, Yunnan Province, China. MycoBank numbers were obtained as described in the MycoBank database (http://www.mycobank.org). In addition, we obtained the habitat data of these three known fungal species from Global Biodiversity Information Facility (www.GBIF.org) (Supplementary Figures 2, 6 and 10).

### DNA extraction, PCR amplification and DNA sequencing

The mycelia of the cultures grown on PDA at room temperature for 8 weeks were used for DNA extraction. The fungal mycelia were scraped off using a sterile scalpel and transferred to 1.5 ml micro-centrifuge tubes under aseptic conditions and kept at −20°C to avoid contaminations until use. DNA extraction was performed on the fungal cultures using Biospin Fungal Genomic DNA Extraction Kit (BioFlux, P.R. China) following the manufacturer’s protocol. To amplify partial gene regions, PCR conditions and primers were set under standard conditions, as shown in Supplementary Table 2. The total volume of PCR mixtures for amplifications was set as described in Tibpromma et al. [17]. Purification and sequencing of PCR products were done by Sangon Biotech Co., Shanghai, China.

### Phylogenetic analyses

ITS and TEF1 sequence data produced in this study were used in BLAST searches in the GenBank database (http://www.blast.ncbi.nlm.nih.gov/) to determine their most probable closely related taxa. The sequence data generated in this study were analysed with closely related taxa retrieved from GenBank and recent publications. The single gene sequence dataset was aligned using the MAFFT v. 7.215 website [18]: (http://mafft.cbrc.jp/alignment/server/index.html) and manually edited in BioEdit v. 7.0 when necessary [19]. The single sequence alignment dataset was combined using BioEdit v.7.0 [19]. The alignment of combined datasets in FASTA format was converted to PHYLIP and NEXUS formats by using the ALignment Transformation EnviRonment (ALTER) website (http://sing.ei.uvigo.es/ALTER/). Phylogenetic trees were run in Randomized Accelerated Maximum Likelihood (RAxML) and Bayesian posterior probabilities (BYPP). ML analysis was performed via the CIPRES Science Gateway (http://www.phylo.org/) [20] using RAxML-HPC BlackBox (8.2.4) section [21] with the general time reversible model (GTR) using a discrete gamma distribution as the evolutionary model. To carry out Bayesian analysis, the model of evolution was estimated using MrModeltest 2.2 [22] as nucleotide substitution models selected for combined datasets. Posterior probabilities (PP) [23] were determined by Markov Chain Monte Carlo sampling (MCMC) in MrBayes v 3.0b4 [24]. The parameters were set as six simultaneous Markov chains and ran for 5000000 generations with sample frequency every 100th generations [25]. The first trees representing the burn-in phase of the analyses (20%) were discarded and the remaining (post-burn) trees were used for calculating posterior probabilities (PP) in the majority rule consensus tree (with the critical value for the topological convergence diagnostic values reaching 0.01) [26].

The phylograms were configured in FigureTree v. 1.4 [27] and reorganized using Microsoft Office PowerPoint 2016, Adobe Acrobat XI Pro, and Adobe Photoshop CC 2019 (Adobe Systems Inc., USA). The sequences generated in this study were submitted to MycoBank and are mentioned under the description part.

### Pairwise homoplasy index

The pairwise homoplasy index (PHI) test was performed by SplitsTree4 to determine the recombination level within phylogenetically closely related species by using the concatenated dataset of closely related species (Supplementary Figures 4 and 8) [28]. Pairwise homoplasy index results lower than 0.05 (Φw < 0.05) indicate the presence of significant recombination in the dataset. The relationships between closely related taxa are visualized by constructing splits graphs from concatenated datasets, using the Log-Det transformation and splits decomposition options.

## Results

### Climatic conditions inside of the cave

The climate inside the cave proved to be highly stable. Climate details of the cavern in which the specimens were collected are provided in Table 1.

**Table 1.** Climatic data inside the cave.

Temperature (°C)	Relative humidity (%)	CO_2_ (ppm)
19.6 ± 0.15	73.9 ± 0.15	1177 ± 2.03

### Phylogenetic analysis of single and combined sequence data

The single and combined datasets of fungi on bats were analysed using maximum likelihood (ML) and Bayesian analyses (BYPP). Both ML and BYPP trees showed similar results in topology and no significant difference was seen (data not presented).

The phylogram of *Fusarium*, generated from RAxML analysis and based on the combined dataset of TUB and TEF1 datasets, showed that our strains grouped together with the *Fusarium incarnatum* clade with relatively high bootstrap support (Supplementary Figure 1).

The *Mortierella* phylogram, generated from RAxML analysis and based on the combined dataset of 28S and ITS datasets, showed that our five isolates separated into three new species (*Mortierella multispora*, *M. rhinolophicola*, and *M. yunnanensis*), which were well separated from other species in the *Mortierella multispora* cluster. Our new species clustered with *M. pisiformis* but were well separated with high bootstrap support (99% ML/ 1 BYPP, Supplementary Figure 3). *Mortierella rhinolophicola* clustered with *M. echinosphaera* and *M. chlamydospora* but were well separated with high bootstrap support (100% ML/ 0.99 BYPP, Supplementary Figure 3). *M. yunnanensis* clustered with *M. amoeboidea* and *M. alpina* but were well separated with high bootstrap support (76% ML/ 0.99 BYPP, Supplementary Figure 3).

The *Mucor* phylogram, generated from RAxML analysis and based on the ITS dataset, showed that our strains grouped together with the *Mucor hiemalis* clade with relatively high bootstrap support (Supplementary Figure 5).

The *Neocosmospora* phylogram, generated from RAxML analysis and based on the combined dataset of LSU, ITS, TEF1, and RPB2 datasets, showed that our new species, *Neocosmospora pallidimors*, was well separated from other *Neocosmospora* species with relatively high bootstrap supports (100% ML/ 1 BYPP, Supplementary Figure 7).

The *Trichoderma* phylogram, generated from RAxML analysis and based on the combined dataset of RPB2 and TEF1 datasets, showed that our strains grouped together with the *Trichoderma harzianum* clade with relatively high bootstrap support (Supplementary Figure 9).

Therefore, we introduce *Mortierella multispora, M. rhinolophicola*, *M. yunnanensis*, and *Neocosmospora pallidimors* as new species. In addition, we report 3 known species: *Fusarium incarnatum, Mucor hiemalis*, and *Trichoderma harzianum* based on evidence from phylogeny and morphology.

### Taxonomy

***Fusarium*** Link, Magazin der Gesellschaft Naturforschenden Freunde Berlin 3 (1): 10 (1809).

The genus *Fusarium* (Hypocreales, Nectriaceae), a filamentous fungus, was introduced by Link [29] with distinctive banana-shaped conidia. This fungus is widely distributed in plants (infecting both monocotyledonous and dicotyledonous) soils, and marine environments [30]. Species within *Fusarium* include the most important plant-pathogenic fungi, causing infections, allergic responses, and acting as pathogens of immunocompromised humans and animals [31]. *Fusarium* species produce remarkable diversity of secondary metabolites and mycotoxins (most notably trichothecenes and fumonisins) that exhibit toxicity to mammal and human health (especially in immunocompromised individuals) [32,33]. Furthermore, members of *Fusarium* have previously been reported in cave systems [11,12].

***Fusarium incarnatum*** (Roberge) Sacc., Sylloge Fungorum 4: 712 (1886).

*MycoBank number:* MB231142; Supplementary Figures 1, 2

*Culture characteristics*: Colonies on PDA (Figure 2) covering 9 cm diam., in 4 weeks at 28°C, cottony, circular, with entire edge, velvety, flossy. Mycelium superficial, at first pink-orange, later becoming yellow-brown; reverse orange-brown, with sporulating and conidiophores and conidial development in culture after 5 months.

*GenBank numbers*: TEF1 = MT024982.

*Notes*: *Fusarium incarnatum* was introduced by Saccardo [34], which belongs to the *F. incarnatum*-*equiseti* species complex. This complex is well-known for containing plant, human, and animal diseases [35] (Supplementary Figure 2). In this study, we found *Fusarium incarnatum*, which was isolated from a bat carcass found in a cave. We assume this fungus can survive in the mild temperature and low oxygen/high carbon dioxide environment of the cave in which they were found. Furthermore, this is the first report of *F. incarnatum* from bat carcasses as well as caves.

***Mortierella*** Coem., Bulletin de l’Académie Royale des Sciences de Belgique Classe des Sciences 15: 536 (1863).

Species of *Mortierella* (Mortierellales, Mortierellaceae) are frequently isolated from soils, dead or dying plant tissues, freshwater, or animal dung samples [15,36,37]. Many show a strong capacity to decompose plant litter, degrade polyaromatic hydrocarbons, and are also potential producers of c-linolenic acid, polyunsaturated fatty acids and arachidonic acid [38,39]. Several species are known to be indirectly beneficial to humans; for example, they are able to degrade herbicidal residues on crop plants; aid mycorrhizal fungi in phosphorus (P) acquisition; and have the ability to synthesize and secrete oxalic acid [40,41]. There have been reports on *Mortierella* species being found in cave systems [11,12].

***Mortierella multispora*** Tibpromma, Karunarathna, Karasaki & Mortimer *sp. nov*.

**Figure 3.**
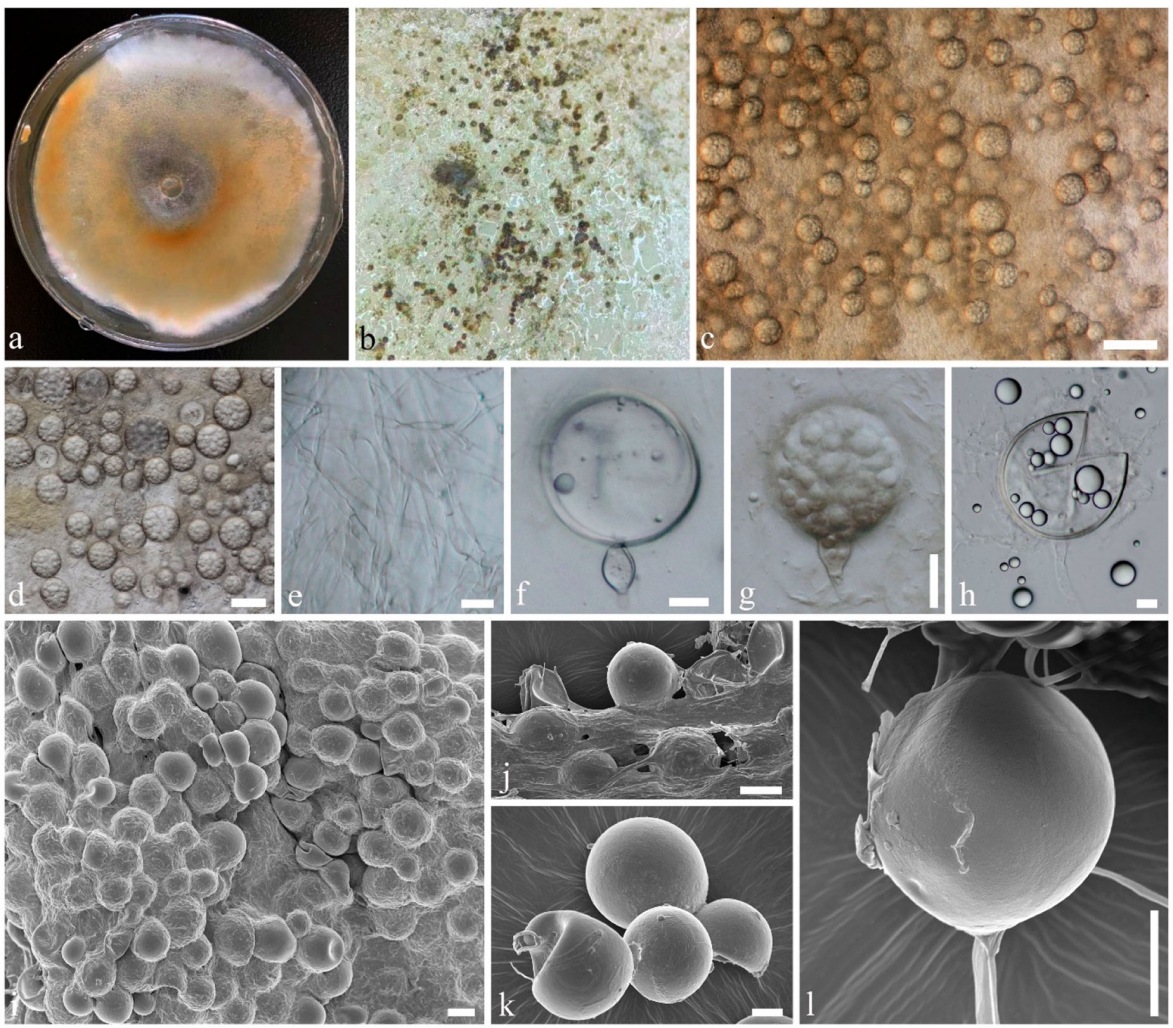
*Mortierella multispora* (KUMCC 20-0005, ex-type). a Colony on PDA, shown from above, grown for 60 days at 28°C. b Sporangia mass form under media. c, d Sporangia. e Mycelia. f, g Sporangia attached with tip of sporangiophores. h Sporangiospores. i–l Sporangia under a SEM. Scale bars: *b* = 50 µm, *c*, *e*, *i*, *j* = 20 µm, *d* = 40 µm, *e* = 2 µm, *f* = 5 µm, *g*, *h*, *k*, *l* = 10 µm.

*MycoBank number:* MB834364; Figure 3; Supplementary Figures 3, 4

*Etymology*: Refers to many different sized spores in sporangia.

*Holotype*: CHINA, Yunnan Province, cave outside of Kunming City, on dead bats (*Rhinolophus affinis*), 5 August 2019, Karasakie S, BatB (YMF1.06174)

*Saprobic or opportunistic pathogen* on bat carcass. **Sexual morph** Undetermined. **Asexual morph** *Sporangiophores* 10–20 µm high (up to 50 µm) × 2–10 µm diam., erect, developed from aerial hyphae and broad at the tip (tip swollen, 5–10 μm wide), hyaline, smooth-walled, non-septate with or without branched, with granules. *Sporangia* 20–50 × 20–50 µm ($\bar{x}$ = 37.5 × 39 µm, *n* = 20), globose to subglobose, 1-celled, unicellular, with multi-spores, hyaline, smooth and thick-walled. *Sporangiospores* 2–12 × 2.5–12 µm ($\bar{x}$ = 6 × 5.58 µm, *n* = 40), globose to subglobose, hyaline, smooth-walled.

*Culture characteristics**:*** Colonies on PDA (Figure 2). Colonies on PDA covering 9 cm diam., in 4 weeks at 28°C, cottony, circular, with entire edge, velvety, flossy. Mycelium superficial, at first white, later becoming yellow-white; reverse yellow-orange, with sporulating and sporangiospores development.

*Material examined:* CHINA, Yunnan Province, cave outside of Kunming City, on dead bats (*Rhinolophus affinis*), 5 August 2019, Karasakie S, BatB (YMF1.06174, **holotype**), ex-type living culture, KUMCC20-0005.

*GenBank numbers*: 28S = MT032146, ITS = MT031921.

*Notes*: *Mortierella multispora* is introduced here with evidence from morphology and phylogeny. Phylogenetic analyses show our new species clusters with *M. pisiformis* with high statistical support (99% in ML, and 1 in PP) (Supplementary Figure 3). Morphological comparisons show that *M. pisiformis* has 25.5–33 μm globose sporangia with 7.5–10 × 12.5–15 μm ellipsoid, pea-shaped, sporangiospores [59], while our new species has 20–50 × 20–50 µm globose to subglobose sporangia with 2–12 × 2.5–12 µm globose to subglobose sporangiospores. Based on a blast search of NCBIs GenBank nucleotide database, the closest hits received using the ITS sequence of our new species are *Mortierella* sp. (GenBank AB542110; Identities = 89.03%) and *Mortierella* sp. (GenBank KP744417; Identities = 90.60%), while closest hits received using the 28S sequence of our new species are *M. lignicola* (GenBank MH868590; Identities = 96.65%), and *M. paraensis* (GenBank NG_042569; Identities = 96.65%).

***Mortierella rhinolophicola*** Tibpromma, Karunarathna, Karasaki & Mortimer *sp. nov*.

**Figure 4.**
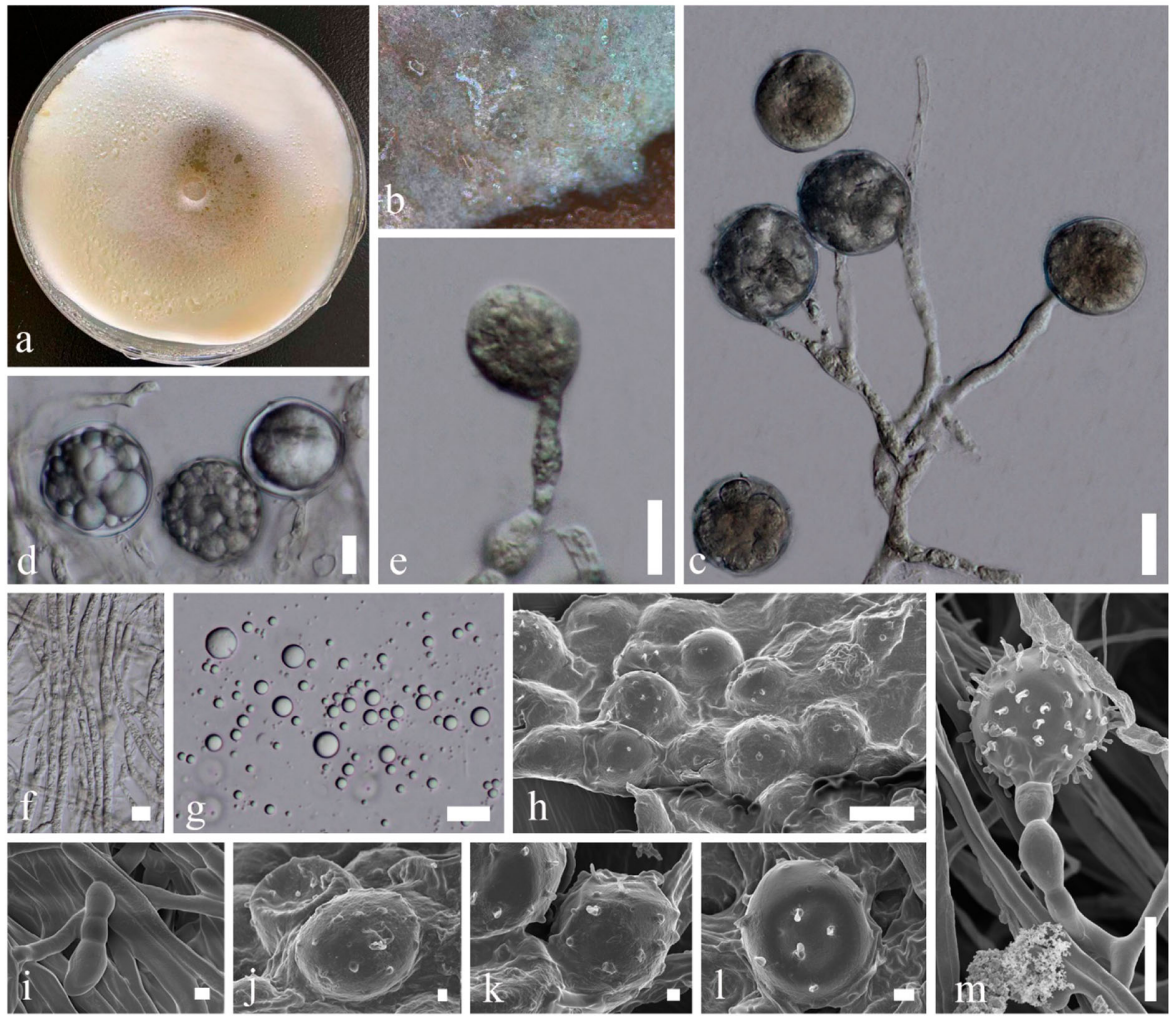
*Mortierella rhinolophicola* (KUMCC 20-0008, ex-type). a Colony on PDA, shown from above, grown for 60 days at 28°C. b Close up mycelium on PDA. c–e Sporangia attached with Sporangiophores. f Mycelia with granules. g Sporangiospores. h, j, k Sporangia under a SEM. i Mycelia under a SEM. m Sporangia attached with sporangiophores under a SEM. Scale bars: c–g, *m* = 10 µm, *h* = 20 µm, *i*, *j*, *k* = 2 µm, *l* = 3 µm.

*MycoBank number:* MB834365; Figure 4; Supplementary Figures 3, 4

*Etymology*: Refer to the host genus *Rhinolophus* (bat)

*Holotype*: CHINA, Yunnan Province, cave outside of Kunming City, on dead bats (*Rhinolophus affinis*), 5 August 2019, Karasakie S, BatE (YMF1.06175)

*Saprobic or opportunistic pathogen* found on bat carcass. **Sexual morph** Undetermined. **Asexual morph** *Sporangiophores* 25–120 µm high, 1–5 µm diam. ($\bar{x}$ = 59.1 × 2.7 µm, *n* = 10), erect with tapering toward the tips, developed from aerial hyphae and simple or branched, hyaline, smooth-walled, non-septate with granules, often branched 2–3 times, swollen some part. *Sporangia* 15–30 × 15–30 µm ($\bar{x}$ = 23.2 × 23.9 µm, *n* = 40), globose to subglobose, 1-celled, unicellular with multi-spores, hyaline, thick-walled with echinulate. *Sporangiospores* 1.5–7 × 1.5–8 µm ($\bar{x}$ = 3.2 × 3.1 µm, *n* = 40), globose to subglobose, hyaline, thick-walled.

*Culture characteristics*: Colonies on PDA (Figure 2). Colonies on PDA covering 9 cm diam., in 4 weeks at 28°C, cottony, circular, with entire edge, velvety, flossy. Mycelium superficial, at first white, later becoming yellow-white; reverse yellow-orange, with sporulating and sporangiospores development.

*Material examined*: CHINA, Yunnan Province, cave outside of Kunming City, on dead bats (*Rhinolophus affinis*), 5 August 2019, Karasakie S, BatE (YMF1.06175, **holotype**), ex-type living culture, KUMCC20-0008; Yunnan Province, cave outside of Kunming City, on dead bats (*Rhinolophus affinis*), 5 August 2019, Karasakie S, BatK (KUMCC20-0014, ex-paratype).

*GenBank numbers*: KUMCC 20-0008: 28S = MT032144, ITS = MT031919; KUMCC 20-0014: 28S = MT032145, ITS = MT031920.

*Notes*: Based on phylogenetic analyses, our new species is well separated from *Mortierella echinosphaera* and *M. chlamydospore* with high statistical support (100% in ML, 0.99 in PP) (Supplementary Figure 3). Our results are consistent with Wagner et al. [14] who mentioned that *M. echinosphaera* and *M. chlamydospora* are closely related. Morphological characteristics show *M. chlamydospora* has globose or elongated chlamydospores, with a varying number of spines that are sometimes smooth when submerged [42,43], and *M. echinosphaera* has globose to elongated, densely spiny chlamydospores [41,42], while our new species has globose to subglobose, echinulate sporangia. A BLAST search of NCBI’s GenBank nucleotide database revealed that the closest hit received for *M. rhinolophicola* (KUMCC 20-0008)*,* using the ITS sequence, was *M. chlamydospora* (GenBank KP881466; Identities = 98.64%), and the closest hit received for *M. rhinolophicola* using the 28S sequence was *M. chlamydospora* (GenBank KP881466; Identities = 97.15%). Based on a BLAST search of NCBI’s GenBank nucleotide database, the closest hit received for *M. rhinolophicola* (KUMCC 20-0014) using the ITS sequence was *M. chlamydospora* (GenBank KP881466.1; Identities = 97.15%) and the closest hits received for *M. rhinolophicola* using the 28S sequence was *M. echinosphaera* (GenBank KC018382; Identities = 98.57%) and *M. chlamydospora* (GenBank HQ667430; Identities = 98.17%).

***Mortierella yunnanensis*** Tibpromma, Karunarathna, Karasaki & Mortimer *sp. nov*.

**Figure 5.**
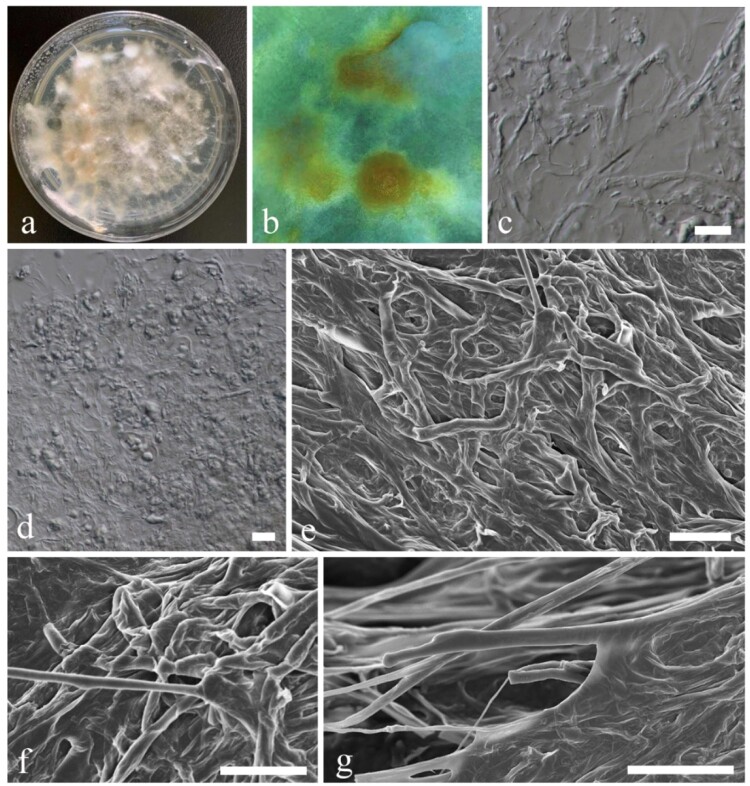
*Mortierella yunnanensis* (KUMCC 20-0009, ex-type). a Colony on PDA shown from above, grown for 60 days at 28°C. b Close up mycelium on PDA form yellow mycelia group. c, d Mycelia under compound microscope. e–g Mycelia under a SEM. Scale bars: *c* = 20 µm, *d*–*g* = 10 µm.

*MycoBank number:* MB834366; Figure 5; Supplementary Figures 3, 4

*Etymology*: refers to the province ‘Yunnan’ where the type species was collected.

*Holotype*: CHINA, Yunnan Province, cave outside of Kunming City, on dead bats (*Rhinolophus affinis*), 5 August 2019, Karasakie S, BatF (YMF1.06176)

*Culture characteristics*: Colonies on PDA (Figure 2). Colonies on PDA covering 9 cm diam., in 4 weeks at 28°C, spreading, with sparse aerial mycelium, irregular, with undulate edge, velvety, flossy. Mycelium superficial, at first white, later becoming yellow-white; reverse yellow-orange, without sporulating on PDA at 6 months. Mycelium hyaline, branch, granules with septate.

*Material examined:* CHINA, Yunnan Province, cave outside of Kunming City, on dead bats (*Rhinolophus affinis*), 5 August 2019, Karasakie S, BatF (YMF1.06176, **holotype**), ex-type living culture, KUMCC20-0009; Yunnan Province, cave outside of Kunming City, on dead bats (*Rhinolophus affinis*), 5 August 2019, Karasakie S, BatJ (KUMCC20-0013, ex-paratype).

*GenBank numbers*: KUMCC 20-0009: LSU = MT032142, ITS = MT031917; KUMCC 20-0013: LSU = MT032143, ITS = MT031918.

*Notes*: Based on phylogenetic analysis, this new species is well separated from other closely related species of *Mortierella* with high statistical support (76% in ML, 0.99 in PP; Supplementary Figure 3). Our new species clustered with *M. amoeboidea*, but our new species did not sporulate in culture in 5 months (Figure 2). In addition, we compared the cultures of *M. amoeboidea* and our new species and found that *M. amoeboidea* colonies are delicate and densely lobed, with some aerial mycelia in the centre, odour faint and sporulation rather poor on all media [44], while our new species shows spreading, with sparse aerial mycelia, velvety and flossy, at first white and later becoming yellow-white. Our morphological data are complemented by the molecular analyses, confirming this as a distinct new species.

Based on a BLAST search of NCBI’s GenBank nucleotide database, the closest hits received for *M. yunnanensis* (KUMCC 20-0009, KUMCC 20-0013) using the ITS sequence were *M. alpina* (GenBank KU738456; Identities = 99.23%) and *M. alpina* (GenBank KU738464; Identities = 99.22%), while the closest hits received for *M. yunnanensis yunnanensis* (KUMCC 20-0009, KUMCC 20-0013) using the 28S sequence were *M. alpina* (GenBank KT699148; Identities = 97.97%) and *M. alpina* (GenBank KC018438; Identities = 97.77%).

***Mucor*** Fresen., Beiträge zur Mykologie 1: 7 (1850)

*Mucor* (Mucorales, Rhizopodaceae) was introduced by Fresenius [45]. Species are saprotrophs that can be easily isolated from soil, dung, water, stored grains, and other plant parts [15,46]. Prior to 2013, this genus had been introduced based solely on morphological characteristics, after which molecular data have been used to resolve mucoralean species [47]. Members of *Mucor* have been reported in caves [11,12].

***Mucor hiemalis*** Wehmer, Annales Mycologici 1 (1): 37 (1903)

*MycoBank number:* MB249401; Supplementary Figures 5, 6

*Culture characteristics*: Colonies on PDA (Figure 2). Colonies on PDA covering 9 cm diam., in 2 weeks at 28°C, spreading, with sparse aerial mycelium with undulate edge, flossy. Mycelium superficial, pale-brown; reverse yellow, without sporulating on PDA at 6 months. Mycelium hyaline, branch, granules with septate.

*GenBank numbers*: ITS: MT020421

*Notes*: *Mucor hiemalis*, a common soil fungus of phylum zygomycota, produces a diverse array of enzymes and lipids. This species has been reported from organic litter in the Suiyang Cave, Henan, China [12]. Desai et al. [48] reported subcutaneous zygomycosis caused by *Mucor hiemalis* in an immunocompromised patient.

***Neocosmospora*** E.F. Sm., Bulletin of the U.S. Department of Agriculture 17: 45 (1899)

*Neocosmospora* (Hypocreales, Nectriaceae) was introduced by Smith [49]. This genus was assigned to the *Fusarium solani* species complex [50]. This genus is widely distributed in soil, plant debris, living plant material, air, and water, in the form of saprobes, plant endophytes, and pathogens, as well as in opportunistic animal pathogens [15]. *Neocosmospora* species have also been reported to be associated with human and animal mycotoxicoses [51]. Importantly, this genus has never been reported from bats or caves.

***Neocosmospora pallidimors*** Tibpromma, Karunarathna, Karasaki & Mortimer *sp. nov*.

**Figure 6.**
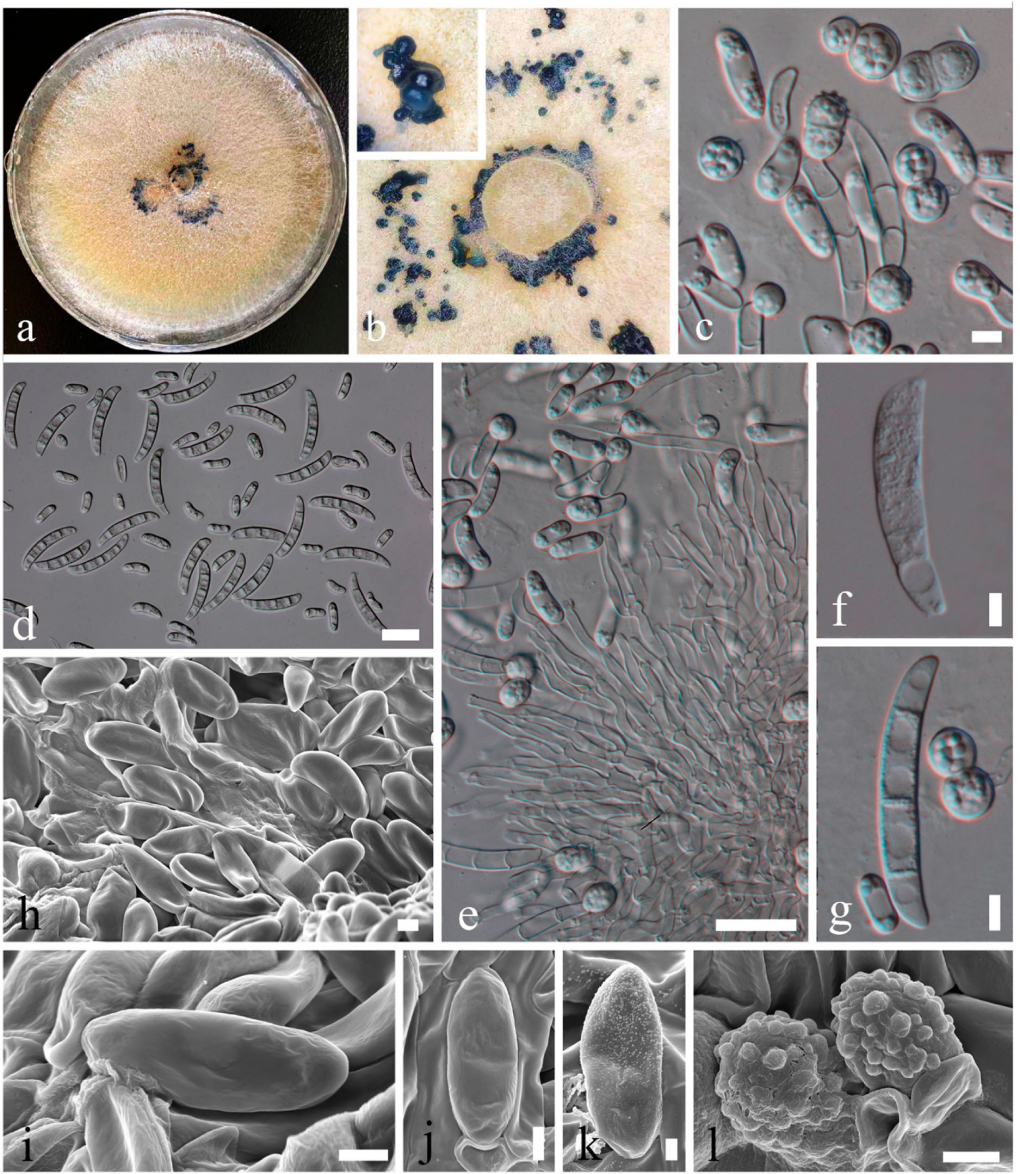
*Neocosmospora pallidimors* (KUMCC 20-0007, ex-type). a Colony on PDA shown from above, grown for 60 days at 28°C. b Close up of mycelium on PDA, showing spore mass (in black). c Aerial microconidia with chlamydospores. d Aerial microconidia with aerial macroconidia. e Aerial conidiophores and phialides. f, g Conidia. h-k Conidia under a SEM. l Chlamydospores. Scale bars: *c*, *f*, *g* = 5 µm, *d*, *e* = 20 µm, *h*–*j*, *l* = 2 µm, *k* = 1 µm.

*MycoBank number:* MB834367; Figure 6; Supplementary Figures 7, 8

*Etymology*: the epithet *pallidimors* referes to ‘pale death’

*Holotype*: CHINA, Yunnan Province, cave outside of Kunming City, on dead bats (*Rhinolophus affinis*), 5 August 2019, Karasakie S, BatD (YMF1.06177)

*Saprobic or opportunistic pathogen* on bat. **Sexual:** Undetermined. **Asexual morph**: *Sporulation* abundant from conidiophores formed directly on the substrate mycelium with rarely conidia on conidiophores. *Conidiophores* 30–70 µm high, 2–4 µm diam., abundant on substrate and aerial mycelium, straight, smooth- and thin-walled, simple or branched, more rarely irregularly or sympodially, bearing terminal and lateral, single monophialides; phialides subcylindrical, subulate to acicular, smooth- and thin-walled, conidiogenous loci with rather inconspicuous periclinal thickening and collarettes; aerial conidia of two types: *microconidia* 5–20 × 3–6 µm ($\bar{x}$ = 6.6 × 6 µm, *n* = 40), oblong to allantoid, 0–1-septate, straight or dorsiventrally curved, base somewhat flattened, granulate, smooth- and thick-walled; *macroconidia* 30–40 × 4–7 µm ($\bar{x}$ = 35.2 × 5.7 µm, *n* = 40), fusiform to lunate, multiseptate, 1–3-septate, straight or dorsiventrally curved, granulate, smooth- and thick-walled. *Chlamydospores* 4–10 × 4–8 µm ($\bar{x}$ = 6.4 × 6.2 µm, *n* = 20), globose to subglobose, septate, often two celled stick together, granulate, rough, echinulate and thick-walled.

*Culture characteristics**:*** Colonies on PDA (Figure 2). Colonies on PDA covering 9 cm diam., in 4 weeks at 28°C, spreading, with sparse aerial mycelium with undulate edge, flossy. Mycelium superficial, yellow-white; reverse yellow, with sporulating on PDA after 4 months.

*Material examined:* CHINA, Yunnan Province, cave outside of Kunming City, on dead bats (*Rhinolophus affinis*), 5 August 2019, Karasakie S, BatD (YMF1.06177, **holotype**), ex-type living culture, KUMCC20-0007; Yunnan Province, cave outside of Kunming City, on dead bats (*Rhinolophus affinis*), 5 August 2019, Karasakie S, BatI (KUMCC20-0012, ex-paratype).

*GenBank numbers*: KUMCC 20-0007: TEF1 = MT024983, LSU = MT032140, ITS = MT031915; KUMCC 20-0012: TEF1 = MT024984, LSU = MT032141, ITS = MT031916.

*Notes*: *Neocosmospora pallidimors* is introduced based on evidence from morphology and phylogeny. This is the first report from bats in a cave. *Neocosmospora pallidimors* is phylogenetically closely related to *N. stercicola* but is well separated with statistical supports (100% in ML, 1 in PP). The morphological characteristics of *N. stercicola* include 0–5-septate conidia with colourless drops of liquid, ovoid to ellipsoidal, single or in chains and pale brown chlamydospores [52], while our new species has 0–3-septate conidia with globose to subglobose, septate, often two-celled rough and echinulate chlamydospores that stick together.

***Trichoderma*** Pers., Neues Magazin für die Botanik 1: 92 (1794)

*Trichoderma* (Hypocreales, Hypocreaceae) was introduced by Persoon [53]. *Trichoderma* forms a large group of microorganisms with global distribution and are known as opportunistic fungi of economic and ecological importance, as they produce toxic secondary metabolites and disease-causing agents of plants, animals, and humans [54]. Several species of *Trichoderma* have been reported in caves [11,12].

***Trichoderma harzianum*** Rifai, Mycological Papers 116: 38 (1969)

*MycoBank number:* MB340299; Supplementary Figures 9, 10.

*Culture characteristics:* Colonies on PDA (Figure 2)

*GenBank numbers*: KUMCC 20-0004: TEF1 = MT024986, RPB2 = MT024987; KUMCC 20-0011: TEF1 = MT024985, RPB2 = MT024988.

*Notes*: *Trichoderma harzianum* is widely recognized as a potential biocontrol agent against several soilborne plant pathogens [55]. This species is reported here for the first time on bats and in caves.

## Discussion

In this paper, we present seven species of fungi – four of them novel – extracted from a pair of bat carcasses in a limestone cave in Yunnan, China. Of these seven species, *Fusarium incarnatum* is known as a plant pathogen [56], and *Neocosmospora pallidimors* belongs to a group known to have highly prevalent and aggressive human and animal fungal pathogens [15], while *Mucor hiemalis* was reported to cause zygomycosis, an opportunistic fungal infection with a high mortality rate infecting across a wide range of substrates, from bread to human skin tissue [48,57]. This density of new species found in an isolated and relatively confined system underscores the exciting potential of subterranea as reservoirs of biological diversity and as a frontier of scientific exploration [9]. This holds particularly true in China, a country thought to contain one of the world’s largest cave-containing limestone karsts systems [58]. This point is further emphasized by the work of Deharveng et al. [59], who reported that 90% of species collected during a caving expedition in Mulun, a National Nature Reserve in Guangxi Province, China, were new to science.

Our study answers the call for further research documenting the relationships – particularly transmission – between fungi and other cave organisms [11]. Discoveries of fungal species on bats are of scientific interest and possible concern, both for humans and for other mammals inhabiting cave systems. The catastrophic effects of *Pseudogymnoascus destructans* on bat hibernacula (e.g. causing white-nose syndrome) triggered a cascade of research on the societal and environmental importance of bats [6, 60]. Difficulties of ecosystem service valuation aside, one well-cited study estimated that a disappearance of North American bats could result in an upward of 3.7 billion dollars in agricultural losses [61]. Prior research on other fungal species within two of the genera we discovered – *Mortierella* and *Neocosmospora* – suggest the potential for economic and ecological implications [51,62]. *Mortiella wolfi* is considered a pathogen solely of animals and is known to induce abortions and cause encephalitis and pneumonia in cattle found in Australia, North America, and Japan [63]. Two reports of human cutaneous infection with *M. wolfii* have also been reported [63,57]. *Neocosmospora* have previously been associated with human and animal mycotoxicoses, and infections are thought to be on the rise [51,62]. A decline in Yunnan bat populations could have devastating effects on the loss of ecosystem services and overall ecosystem health. Further research is necessary to understand how bats may act as carriers for these fungi as well as on the ecological and economic implications for such symbiotic relationships.

Our study presents not only new species of pathogens with unknown pathogenicities but also new sources of already-known pathogens with the potential to affect both crops and mammal populations. Further studies investigating more cave systems across a broader region are still required, and this work needs to be coupled with biomedical studies investigating the pathogenicity of these newly discovered species.

## Supplementary Material

Supplemental Material

## References

[1] Trajano E. Ecological classification of subterranean organisms. Encyclopedia of caves, Vol. 1. Amsterdam: Elsevier Academic Press; 2012. p. 275–277.

[2] Culver DC, Sket B. Hotspots of subterranean biodiversity in caves and wells. J Cave Karst Stud. 2000;62(1):11–17.

[3] Jurado V, Laiz L, Rodriguez-Nava V, et al. Pathogenic and opportunistic microorganisms in caves. Int J Speleol. 2010;39(1):2.

[4] Igreja RP. Infectious diseases associated with caves. Wilderness Environ Med. 2011;22(2):115–121. 21664559 10.1016/j.wem.2011.02.012

[5] Fisher MC, Henk DA, Briggs CJ, et al. Emerging fungal threats to animal, plant and ecosystem health. Nature. 2012;484(7393):186–194. 22498624 10.1038/nature10947 PMC3821985

[6] Frick WF, Pollock JF, Hicks AC, et al. An emerging disease causes regional population collapse of a common North American bat species. Science. 2010;329(5992):679–682. 20689016 10.1126/science.1188594

[7] Puechmaille SJ, Frick WF, Kunz TH, et al. White-nose syndrome: is this emerging disease a threat to European bats? Trends Ecol. Evol. (Amst.). 2011;26(11):570–576. 10.1016/j.tree.2011.06.013 21835492

[8] McLeod DS, Mortimer RH, Perry-Keene DA, et al. Histoplasmosis in Australia: report of 16 cases and literature review. Medicine (Baltimore). 2011;90(1):61–68. 21200187 10.1097/MD.0b013e318206e499

[9] Kambesis P. The importance of cave exploration to scientific research. J Cave Karst Stud. 2007;69(1):46–58.

[10] Dobat K. Ein bisherunveroffentlichtesbotanischesmanuskript Alexander von Humboldts: Plantae subterranaeEurop. 1794 cum Iconibus. Akademie Der Wissenschaften und der Literatur. 1967;6:16–19.

[11] Vanderwolf KJ, Malloch D, McAlpine DF, et al. A world review of fungi, yeasts, and slime molds in caves. Int J Speleol. 2013;42(1):9.

[12] Zhang ZF, Liu F, Zhou X, et al. Culturable mycobiota from Karst caves in China, with descriptions of 20 new species. Pers: Mol Phylogeny Evol Fungi. 2017;39:1. 10.3767/persoonia.2017.39.01 PMC5832949 29503468

[13] Zhang ZF, Zhao P, Cai L. Origin of cave fungi. Front Microbiol. 2018;9:1407. 30013527 10.3389/fmicb.2018.01407 PMC6036247

[14] Wagner L, Stielow B, Hoffmann K, et al. A comprehensive molecular phylogeny of the Mortierellales (Mortierellomycotina) based on nuclear ribosomal DNA. Pers: Mol Phylogeny Evol Fungi. 2013;30:77–93. 10.3767/003158513X666268 PMC3734968 24027348

[15] Sandoval-Denis M, Lombard L, Crous PW. Back to the roots: a reappraisal of *Neocosmospora*. Pers-Mol Phylogeny Evol Fungi. 2019;43:90–185. 10.3767/persoonia.2019.43.04 PMC7085857 32214499

[16] Tibpromma S, Mortimer PE, Karunarathna SC, et al. Morphology and multi-gene phylogeny Reveal *Pestalotiopsispinicola* sp. nov. and a New host Record of *Cladosporium anthropophilum* from Edible Pine (Pinus armandii) Seeds in Yunnan Province, China. Pathogens. 2019;8(4):285. 31817121 10.3390/pathogens8040285 PMC6963873

[17] Tibpromma S, Hyde KD, McKenzie EH, et al. Fungal diversity notes 840–928: micro-fungi associated with Pandanaceae. Fungal Divers. 2018;93(1):1–160.

[18] Katoh K, Standley DM. A simple method to control over-alignment in the MAFFT multiple sequence alignment program. Bioinformatics. 2016;32(13):1933–1942. 27153688 10.1093/bioinformatics/btw108 PMC4920119

[19] Hall T. BioEdit version 7.0. 2004 http://www.mbio–ncsuedu/bioedit/bioedit.html.

[20] Miller MA, Pfeiffer W, Schwartz T. Creating the CIPRES Science Gateway for inference of large phylogenetic trees. In 2010 gateway computing environments workshop (GCE) 2010;14:1–8.

[21] Stamatakis A. RAxML-VI-HPC: maximum likelihood-based phylogenetic analyses with thousands of taxa and mixed models. Bioinformatics. 2006;22(21):2688–2690. 16928733 10.1093/bioinformatics/btl446

[22] Nylander JA. MrModeltest 2.0. Program distributed by the author. Evolutionary Biology Centre, Uppsala University. Available at http://www.ebc.uu.se/systzeo/staff/nylander.html. 2004.

[23] Rannala B, Yang Z. Probability distribution of molecular evolutionary trees: a new method of phylogenetic inference. J Mol Evol. 1996;43(3):304–311. 8703097 10.1007/BF02338839

[24] Ronquist F, Huelsenbeck JP. Mrbayes 3: Bayesian phylogenetic inference under mixed models. Bioinformatics. 2003;19(12):1572–1574. 12912839 10.1093/bioinformatics/btg180

[25] Cai L, Jeewon R, Hyde KD. Phylogenetic investigations of Sordariaceae based on multiple gene sequences and morphology. Mycol Res. 2006;110(2):137–150. 16378718 10.1016/j.mycres.2005.09.014

[26] Maharachchikumbura SS, Hyde KD, Jones EG, et al. Towards a natural classification and backbone tree for Sordariomycetes. Fungal Divers. 2015;72(1):199–301.

[27] Rambaut A. Fig. Tree. Tree figure drawing tool. Version 1.4. 0. University of Edinburgh: Institute of Evolutionary Biology [online]. 2006. Available: bio. ed. ac. uk/.

[28] Huson DH. Splitstree: analyzing and visualizing evolutionary data. Bioinformatics. 1998;14(1):68–73. 9520503 10.1093/bioinformatics/14.1.68

[29] Link HF. Observationes in ordines plantarum naturales. Dissertatio I. Mag GesNaturf Freunde Berlin. 1809;3:3–42.

[30] Armstrong GM. Armstrong GM A, Armstrong JK A. Formaespeciales and races of *Fusarium oxysporum* causing wilt diseases. 1981.

[31] Maryani N, Sandoval-Denis M, Lombard L, et al. New endemic *Fusarium* species hitch-hiking with pathogenic *Fusarium* strains causing Panama disease in small-holder banana plots in Indonesia. Persoonia-Molecular Phylogeny and Evolution of Fungi. 2019;43(1):48–69. 10.3767/persoonia.2019.43.02 PMC7085855 32214497

[32] O’Donnell K, Ward TJ, Geiser DM, et al. Genealogical concordance between the mating type locus and seven other nuclear genes supports formal recognition of nine phylogenetically distinct species within the *Fusarium graminearum* clade. Fungal Genet Biol. 2004;41(6):600–623. 15121083 10.1016/j.fgb.2004.03.003

[33] O’Donnell K, McCormick SP, Busman M, et al. ‘Toxigenic *Fusarium* species: Identity and mycotoxicology’ revisited. Mycologia. 2018;110(6):1058–1080. 30481135 10.1080/00275514.2018.1519773

[34] Saccardo PA. Syllogehyphomycetum. Syllogefungorum. 1886;4:1–807.

[35] Jacobs A, Mojela L, Summerell B, et al. Characterisation of members of the *Fusarium incarnatum-equiseti* species complex from undisturbed soils in South Africa. Antonie van Leeuwenhoek. 2018;111(11):1999–2008. 29777450 10.1007/s10482-018-1093-x

[36] Hyde KD, Hongsanan S, Jeewon R, et al. Fungal diversity notes 367–490: taxonomic and phylogenetic contributions to fungal taxa. Fungal Divers. 2016;80(1):1–270.

[37] Nguyen TT, Park SW, Pangging M, et al. Molecular and morphological Confirmation of three Undescribed species of *Mortierella* from Korea. Mycobiology. 2019;47(1):31–39. 30988989 10.1080/12298093.2018.1551854 PMC6450579

[38] Ogawa J, Sakuradani E, Kishino S, et al. Polyunsaturated fatty acids production and transformation by *Mortierellaalpina* and anaerobic bacteria. Eur J Lipid Sci Technol. 2012;114(10):1107–1113.

[39] Ellegaard-Jensen L, Aamand J, Kragelund BB, et al. Strains of the soil fungus *Mortierella* show different degradation potentials for the phenylurea herbicide diuron. Biodegradation. 2013;24(6):765–774. 23361127 10.1007/s10532-013-9624-7

[40] Osorio NW, Habte M. Soil phosphate desorption induced by a phosphate-solubilizing fungus. Commun Soil Sci Plant Anal. 2014;45(4):451–460.

[41] Tamayo-Vélez Á, Osorio NW. Soil fertility improvement by litter decomposition and inoculation with the fungus *Mortierella* sp. in avocado plantations of Colombia. Commun Soil Sci Plant Anal. 2018;49(2):139–147.

[42] Chesters CG. *Azygozygumchlamydosporum*nov. gen. et sp. A phycomycete associated with a diseased condition of *Antirrhinum majus*. Trans Br Mycol Soc. 1933;18(3):199–IN3.

[43] Plaats-Niterink AJ VD, Samson RA, Stalpers JA, et al. Some Oomycetes and Zygomycetes with asexual echinulate reproductive structures. Pers-Mol Phylogeny Evol Fungi. 1976;9(1):85–93.

[44] Gams W. Some new or noteworthy species of Mortierella. Pers-Mol Phylogeny Evol Fungi. 1976;9(1):111–140.

[45] Fresenius G. Beiträgezurmykologie 1850;1:1–38.

[46] Baldin C, Ibrahim AS. Molecular mechanisms of mucormycosis—the bitter and the sweet. PLoS Pathog. 2017;13(8):e1006408. 28771587 10.1371/journal.ppat.1006408 PMC5542377

[47] Hoffmann K, Pawłowska J, Walther G, et al. The family structure of the Mucorales: a synoptic revision based on comprehensive multigene-genealogies. Pers: Mol Phylogeny Evol Fungi. 2013;30:57. 10.3767/003158513X666259 PMC3734967 24027347

[48] Desai RP, Joseph NM, Ananthakrishnan N, et al. Subcutaneous zygomycosis caused by *Mucor hiemalis* in an immunocompetent patient. Australas Med J. 2013;6(7):374. 23940499 10.4066/AMJ.2013.1764 PMC3737762

[49] Smith EF. Wilt disease of cotton, watermelon and cowpea (*Neocosmospora*nov. gen.). US. Dep. Agric. Div. Veg. Physiol. Pathol. Bull. 1899;(17):1–54.

[50] O’Donnell K. Molecular phylogeny of the *Nectriahaematococca-Fusarium solani* species complex. Mycologia. 2000;92(5):919–938.

[51] Antonissen G, Martel A, Pasmans F, et al. The impact of *Fusarium* mycotoxins on human and animal host susceptibility to infectious diseases. Toxins (Basel). 2014;6(2):430–452. 24476707 10.3390/toxins6020430 PMC3942744

[52] Šišić A, Al-Hatmi AM, Baćanović-Šišić J, et al. Two new species of the *Fusarium solani* species complex isolated from compost and hibiscus (*Hibiscus* sp.). Antonie van Leeuwenhoek. 2018;111(10):1785–1805. 29569107 10.1007/s10482-018-1068-y

[53] Persoon CH, Persoon-Deen C. Neuerversuch; einersystematischeneintheilung der schwämme. 1794.

[54] Dou K, Lu Z, Wu Q, et al. A novel polyphasic identification system for genus *Trichoderma*. bioRxiv. 2018;481382.

[55] Papavizas GC. *Trichoderma* and *Gliocladium*: biology, ecology, and potential for biocontrol. Annu Rev Phytopathol. 1985;23(1):23–54.

[56] Dastjerdi R, Karlovsky P. Systemic infection of maize, sorghum, rice, and beet seedlings with fumonisin-producing and nonproducing *Fusarium verticillioides* strains. Plant Pathol. J.. 2015;31(4):334. 26672472 10.5423/PPJ.OA.05.2015.0088 PMC4677742

[57] Ribes JA, Vanover-Sams CL, Baker DJ. Zygomycetes in human disease. Clin Microbiol Rev. 2000;13(2):236–301. 10756000 10.1128/cmr.13.2.236-301.2000 PMC100153

[58] Whitten T. Applying ecology for cave management in China and neighbouring countries. J Appl Ecol. 2009;46(3):520–523.

[59] Deharveng L, Brehier F, Bedos A. The Mulun karst, a hot–spot of subterranean biodiversity in China. Paper presented at the 19th International Symposium of Subterranean Biology, Fremantle, Western Australia, 22–26 September 2008.

[60] Minnis AM, Lindner DL. Phylogenetic evaluation of Geomyces and allies reveals no close relatives of *Pseudogymnoascus destructans*, comb. nov., in bat hibernacula of eastern North America. Fungal Biol. 2013;117(9):638–649. 24012303 10.1016/j.funbio.2013.07.001

[61] Boyles JG, Cryan PM, McCracken GF, et al. Economic importance of bats in agriculture. Science. 2011;332(6025):41–42. 21454775 10.1126/science.1201366

[62] Sutton DA, Brandt ME. *Fusarium* and other opportunistic hyaline fungi. In Versalovic J, Carroll K, Funke G, Jorgensen JH, Landry ML, editors. Manual of clinical microbiology. 10th ed. Washington, DC: ASM Press; 2011 Jan 1. p. 1853–1879.

[63] Layios N, Canivet JL, Baron F, et al. *Mortierellawolfii*–associated invasive disease. Emerging Infect. Dis.. 2014;20(9):1591. 10.3201/eid2009.140469 PMC4178392 25153198

