# Supplementary material for: Discovery of novel fungal species and pathogens on bat carcasses in a cave in Yunnan Province, China
Source: Emerg Microbes Infect. 2020 Jul 9;9(1):1554–66. doi: 10.1080/22221751.2020.1785333 (PMC7473127; doi:10.1080/22221751.2020.1785333)
Supplement: Supplemental Material [file TEMI_A_1785333_SM2640.zip › Supplementary files/Supplementary_figures.docx]

**Supplementary figures 1-10**


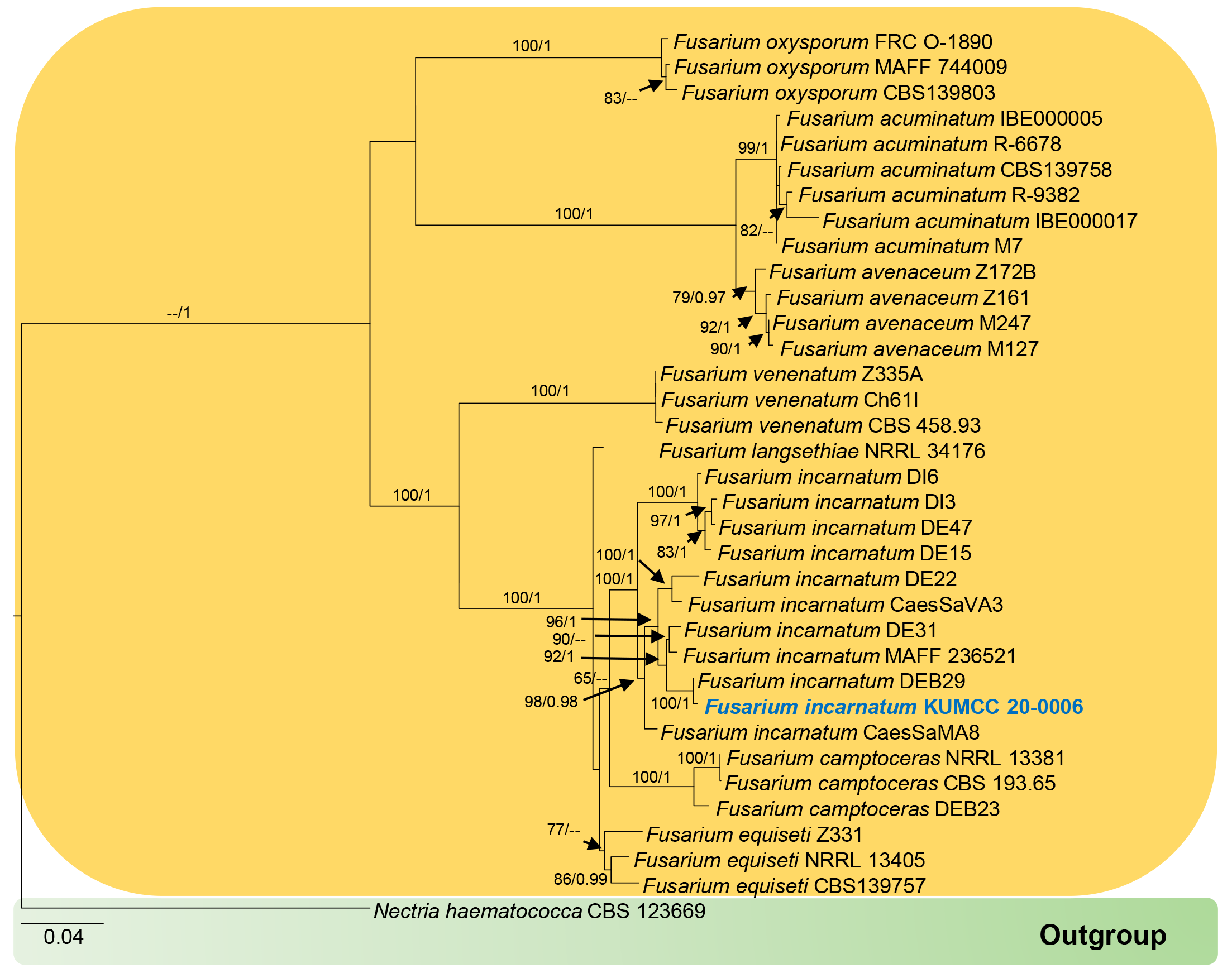


**Supplementary figure 1.** **Phylogram generated from RAxML analysis based on combined β-tubulin and TEF1 sequence data.** There were 38 strains included in the combined sequence analysis, which comprise 1688 characters with gaps. *Nectria haematococca* (CBS 123669) was used as the outgroup taxon. Tree topology of the ML analysis was similar to the BYPP. The best scoring RAxML tree with a final likelihood value of -7706.764776 is presented. The matrix had 753 distinct alignment patterns, with 22.69% undetermined characters or gaps. Estimated base frequencies were as follows: A = 0.216014, C = 0.296433, G = 0.230486, T = 0.257066; substitution rates AC = 1.105912, AG = 3.527327, AT = 1.624501, CG = 0.710313, CT = 5.739681, GT = 1.000000; gamma distribution shape parameter a = 0.358672. Bootstrap support values for ML equal to or greater than 60% and BYPP from MCMC analyses equal to or greater than 0.95 are given above/below the nodes. The ex-type strains are indicated in bold. Newly generated sequences are indicated in blue.


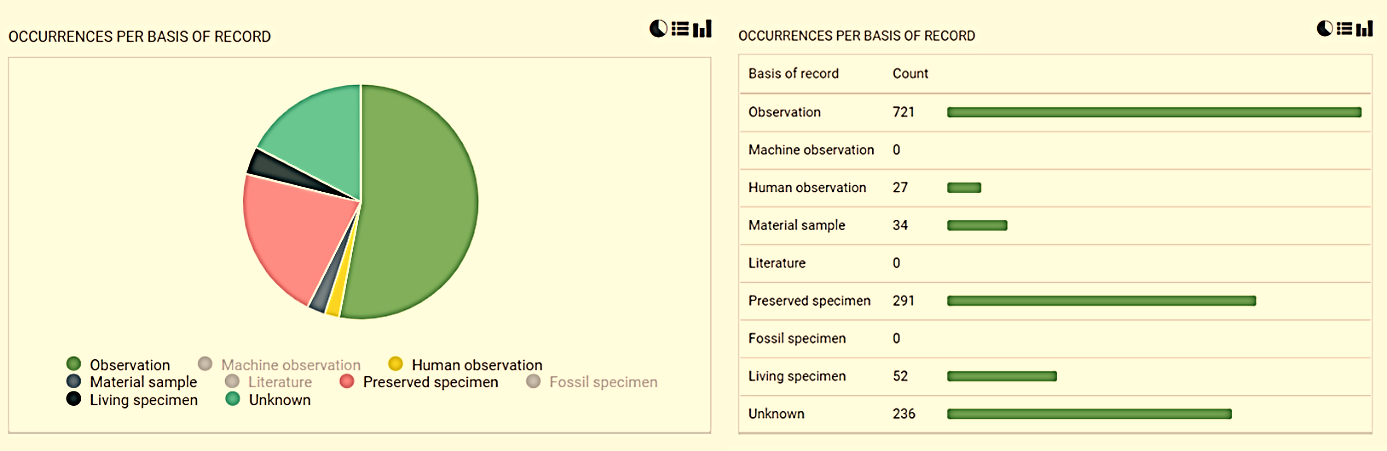
 **Supplementary figure 2. *Fusarium incarnatum* occurrences during 1900-2017 with 1,361 records obtained from the Global Biodiversity Information Facility (accessed 7 March 2020).**


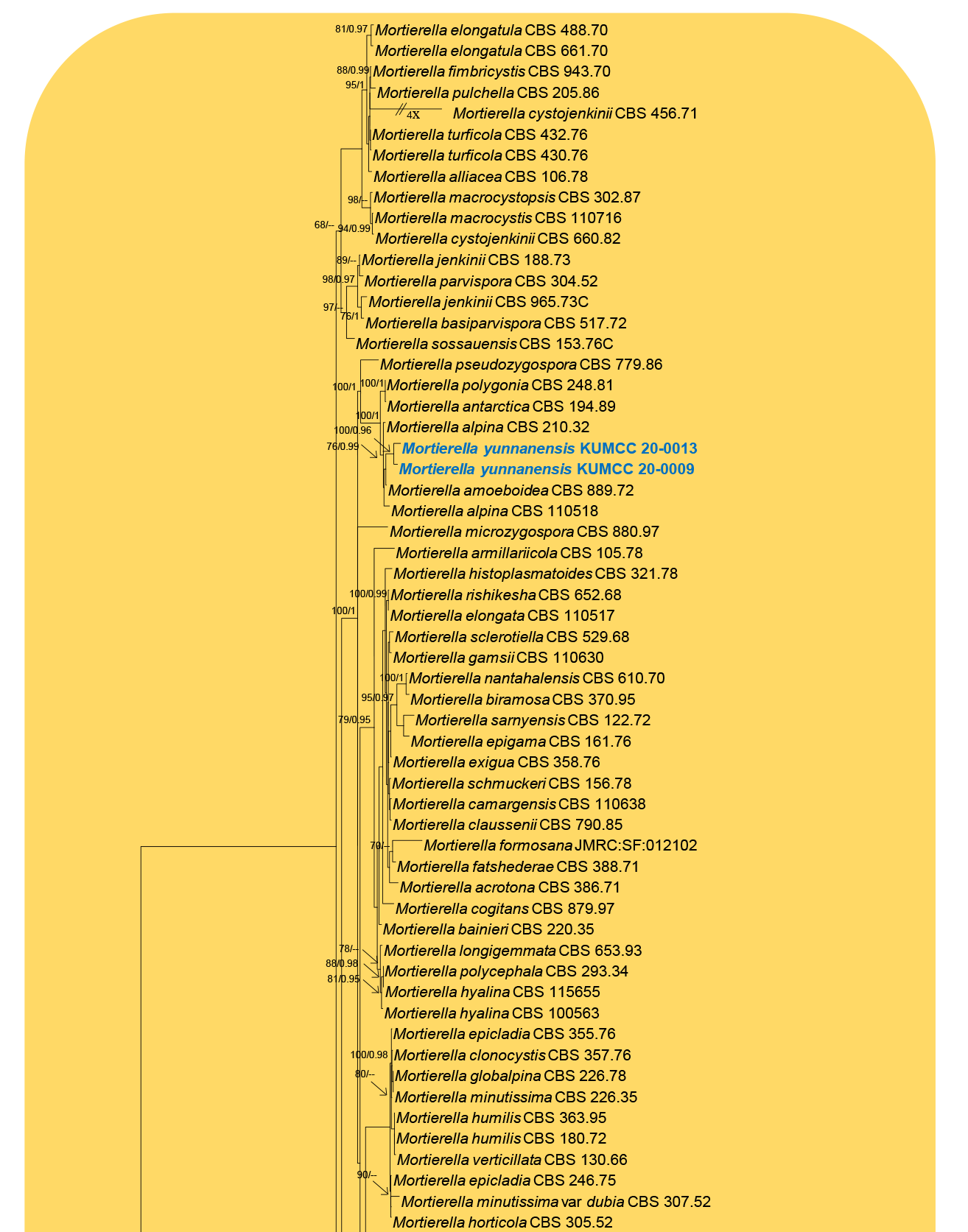


**Supplementary figure 3.** **Phylogram generated from RAxML analysis based on combined 28S and ITS sequence data.** There were 111 strains included in the combined sequence analysis, which comprise 2098 characters with gaps. *Umbelopsis isabellina* (NRRL 1757) was used as the outgroup taxon. Tree topology of the ML analysis was similar to the BYPP. The best scoring RAxML tree with a final likelihood value of -26502.386953 is presented. The matrix had 1366 distinct alignment patterns, with 35.21% undetermined characters or gaps. Estimated base frequencies were as follows: A = 0.277000, C = 0.185529, G = 0.245279, T = 0.292192; substitution rates AC = 1.369595, AG = 3.400308, AT = 1.785912, CG = 0.829085, CT = 5.122194, GT = 1.000000; gamma distribution shape parameter a = 0.305101. Bootstrap support values for ML equal to or greater than 60% and BYPP from MCMC analyses equal to or greater than 0.95 are given above/below the nodes. The ex-type strains are indicated in bold. Newly generated sequences are indicated in blue.


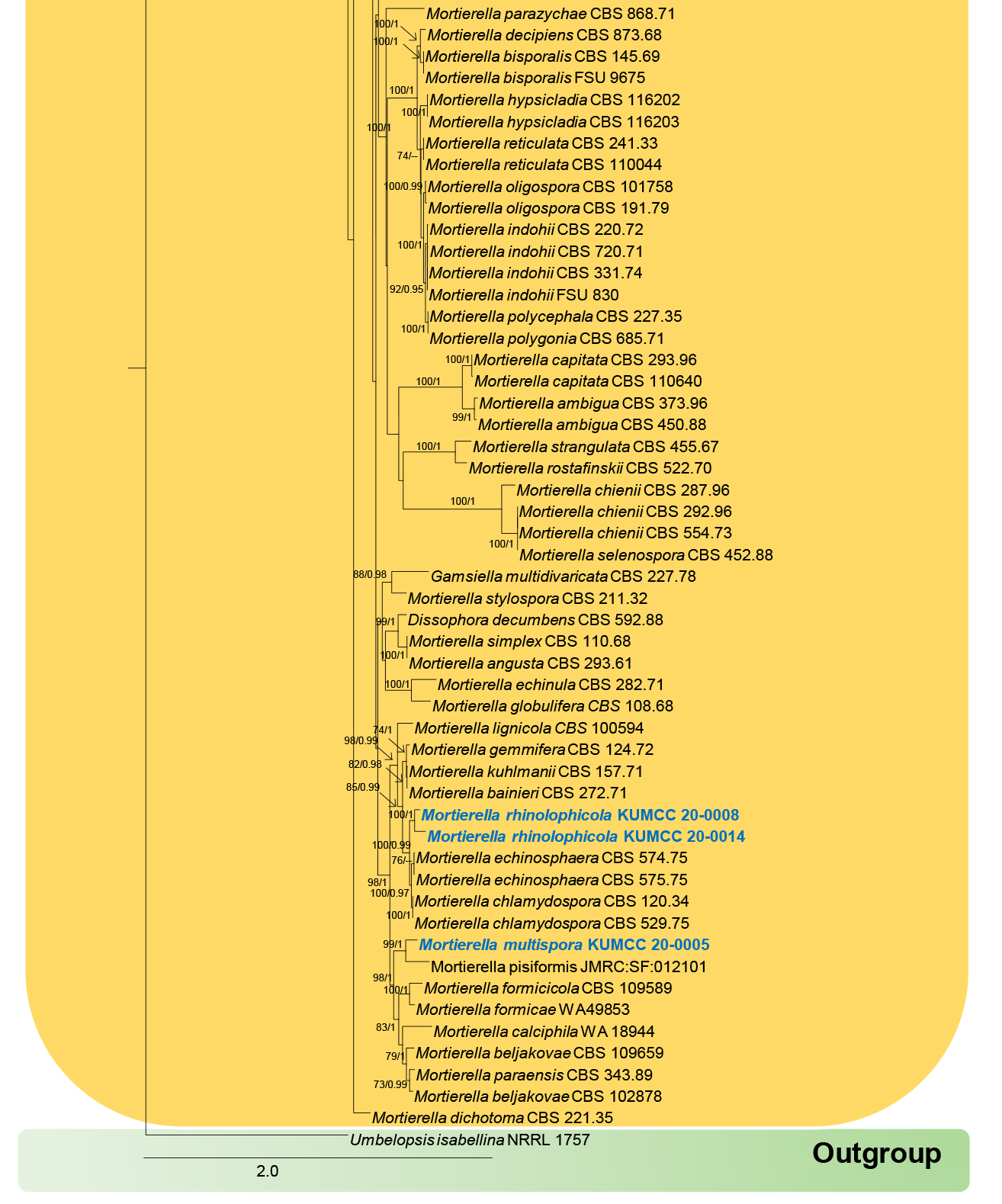


**Supplementary figure 3.** **(cont.)**


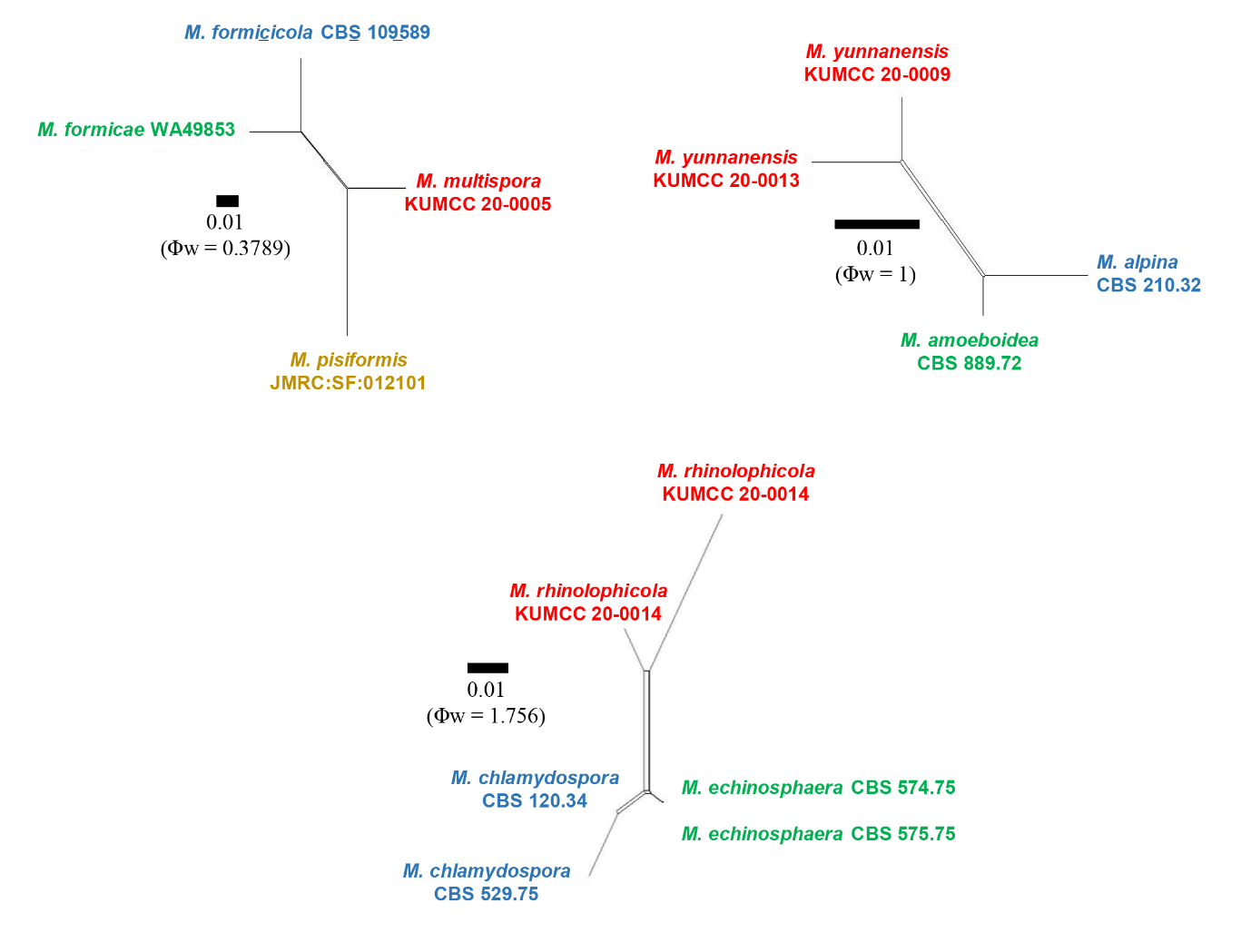


**Supplementary figure 4. Split graphs showing the results of the pairwise homoplasy index (PHI) tests of closely related taxa using LogDet transformation and splits decomposition.** PHI test results (Φw) ≤ 0.05 indicate significant recombination within the dataset.


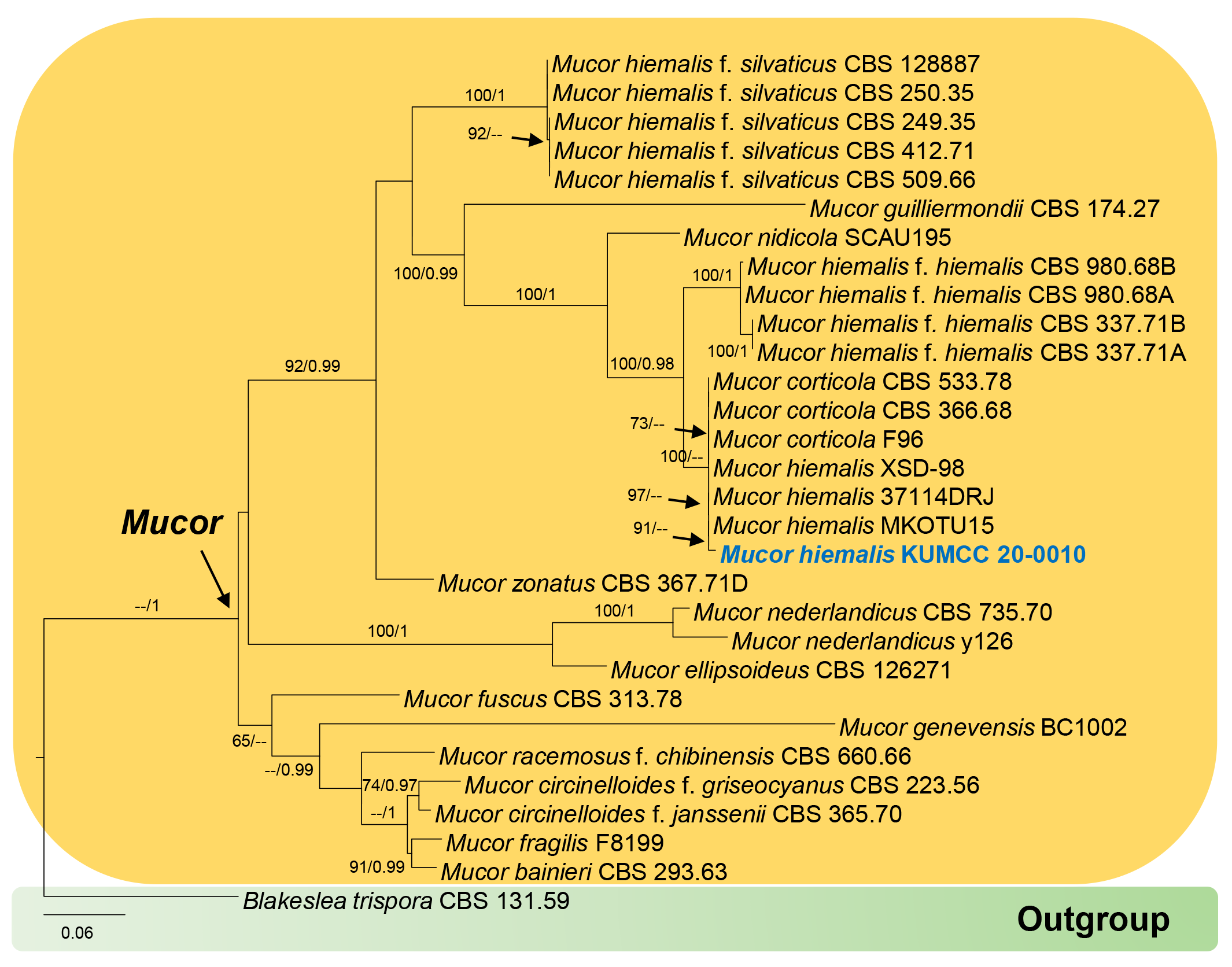


**Supplementary figure 5.** **Phylogram generated from RAxML analysis based on ITS sequence data.** There were 30 strains included in the combined sequence analysis, which comprise 714 characters with gaps. *Blakeslea trispora* (CBS 131.59) was used as the outgroup taxon. Tree topology of the ML analysis was similar to the BYPP. The best scoring RAxML tree with a final likelihood value of -4616.043768 is presented. The matrix had 379 distinct alignment patterns, with 13.58% undetermined characters or gaps. Estimated base frequencies were as follows: A = 0.310501, C = 0.174886, G = 0.182936, T = 0.331677; substitution rates AC = 0.849204, AG = 2.322529, AT = 1.203698, CG = 0.341306, CT = 2.943074, GT = 1.000000; gamma distribution shape parameter a = 0.339651. Bootstrap support values for ML equal to or greater than 60% and BYPP from MCMC analyses equal to or greater than 0.95 are given above/below the nodes. The ex-type strains are indicated in bold. Newly generated sequences are indicated in blue.


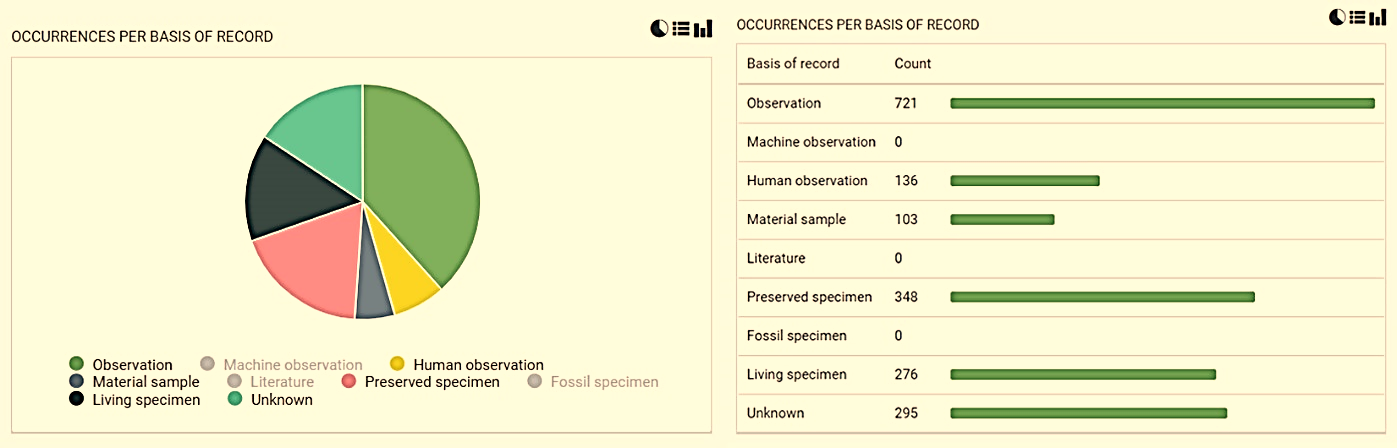


**Supplementary figure 6. *Mucor hiemalis* occurrences during 1900-2015 with 518 records was obtained from Global Biodiversity Information Facility (accessed 7 March 2020).**


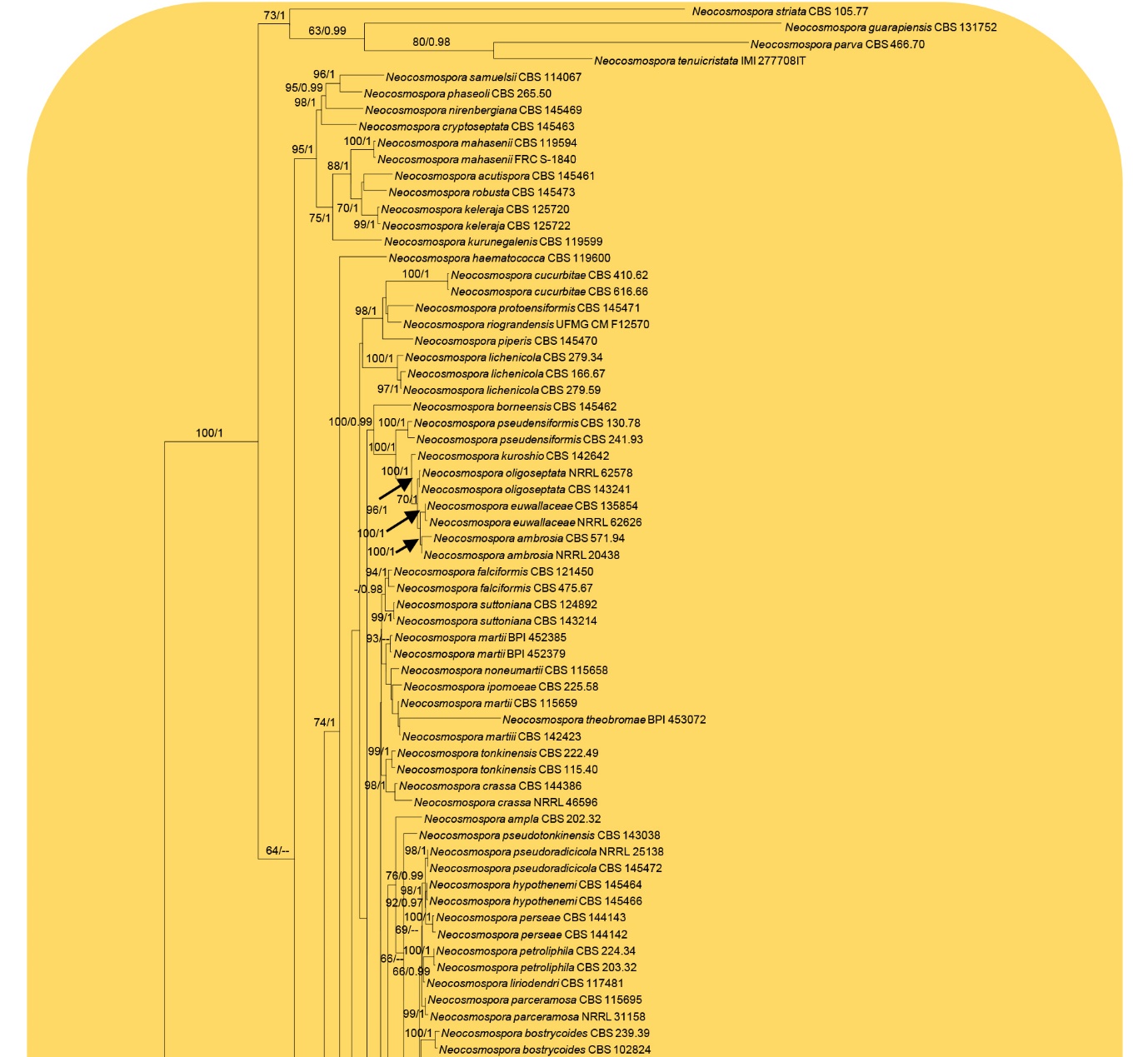


**Supplementary figure 7.** **Phylogram generated from RAxML analysis based on combined on LSU, ITS, RPB2 and TEF1 sequence data.** There were 114 strains included in the combined sequence analysis, which comprise 4275 characters with gaps. *Geejayessia atrofusca* (NRRL 22316) and *Geejayessia cicatricum* (CBS 125552) were used as the outgroup taxon. Tree topology of the ML analysis was similar to the BYPP. The best scoring RAxML tree with a final likelihood value of -33463.712041 is presented. The matrix had 1942 distinct alignment patterns, with 37.25% undetermined characters or gaps. Estimated base frequencies were as follows: A = 0.239957, C = 0.275455, G = 0.261472, T = 0.223116; substitution rates AC = 1.959271, AG = 4.695377, AT = 2.047385, CG = 1.275658, CT = 10.465598, GT = 1.000000; gamma distribution shape parameter a = 0.286746. Bootstrap support values for ML equal to or greater than 60% and BYPP from MCMC analyses equal to or greater than 0.95 are given above/below the nodes. The ex-type strains are indicated in bold. Newly generated sequences are indicated in blue.


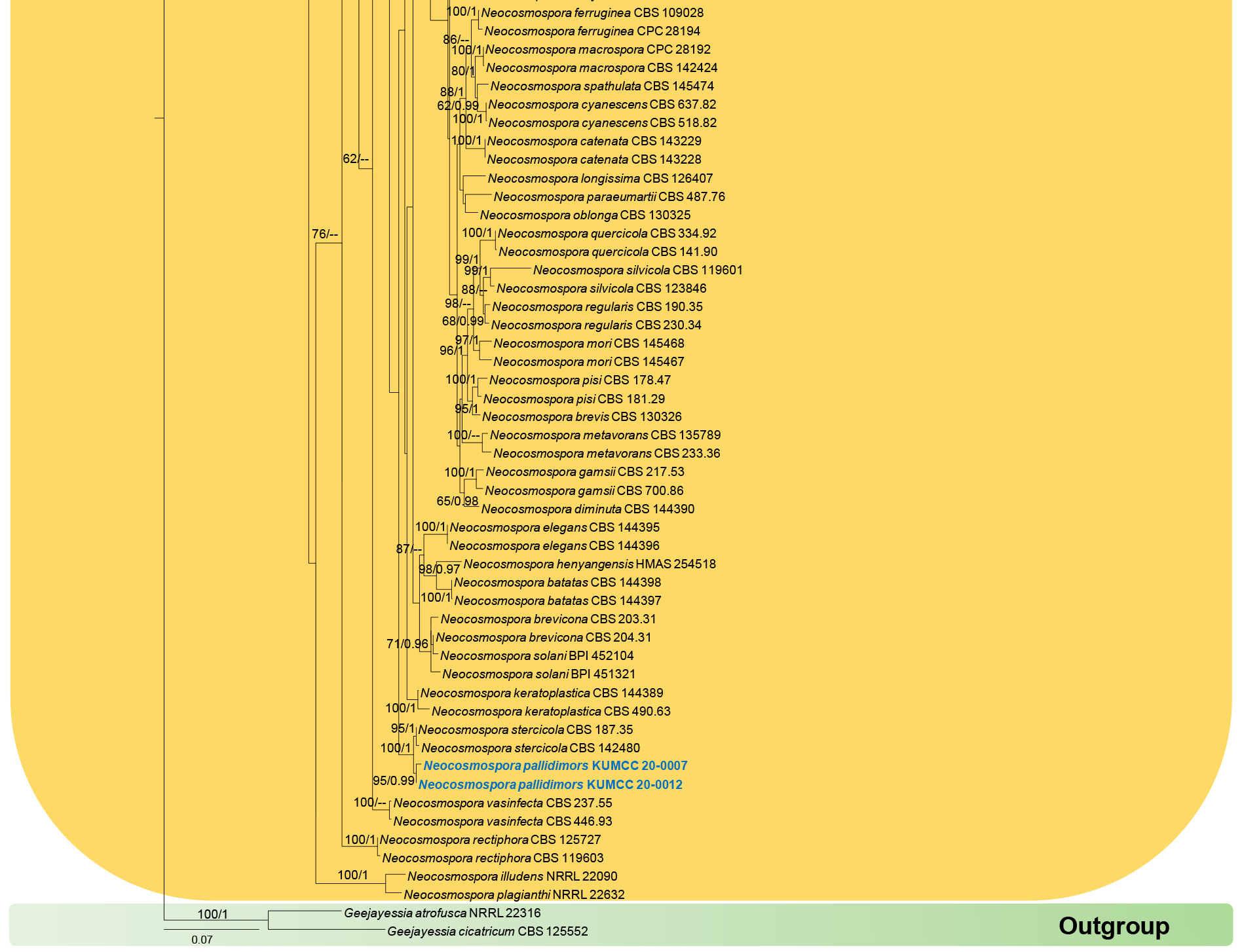


**Supplementary figure 7. (cont.)**


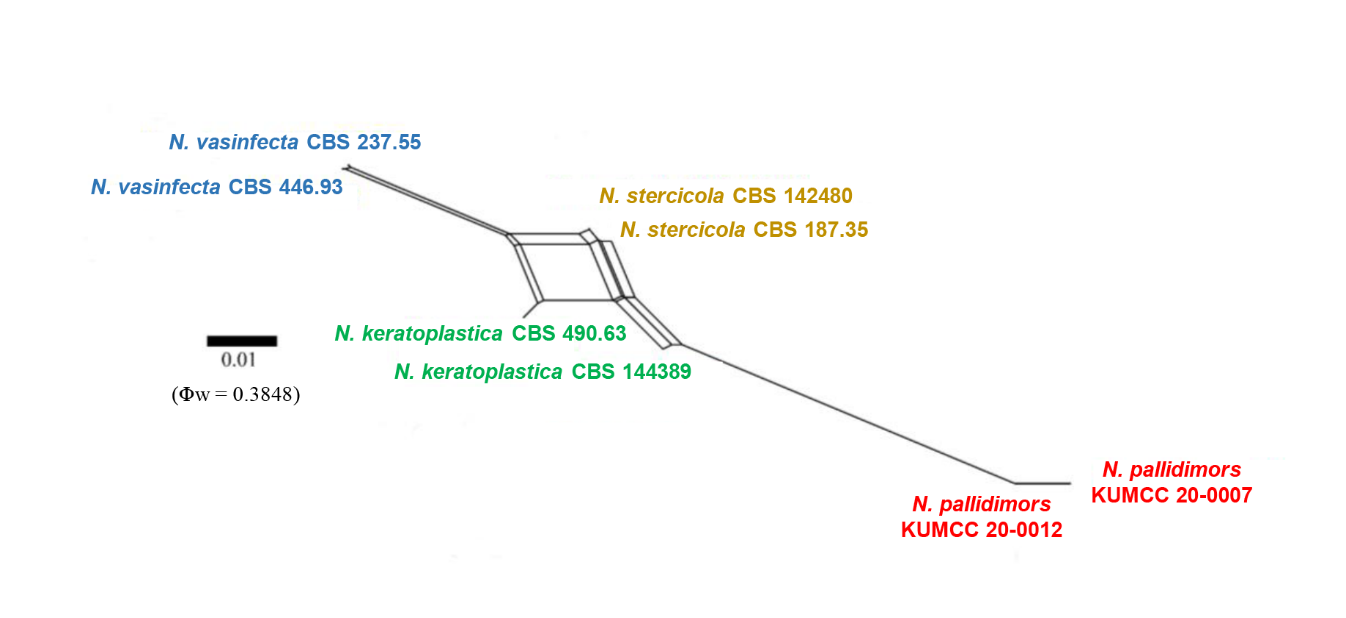


**Supplementary figure 8. Split graphs showing the results of the pairwise homoplasy index (PHI) tests of closely related taxa using LogDet transformation and splits decomposition.** PHI test results (Φw) ≤ 0.05 indicate significant recombination within the dataset.


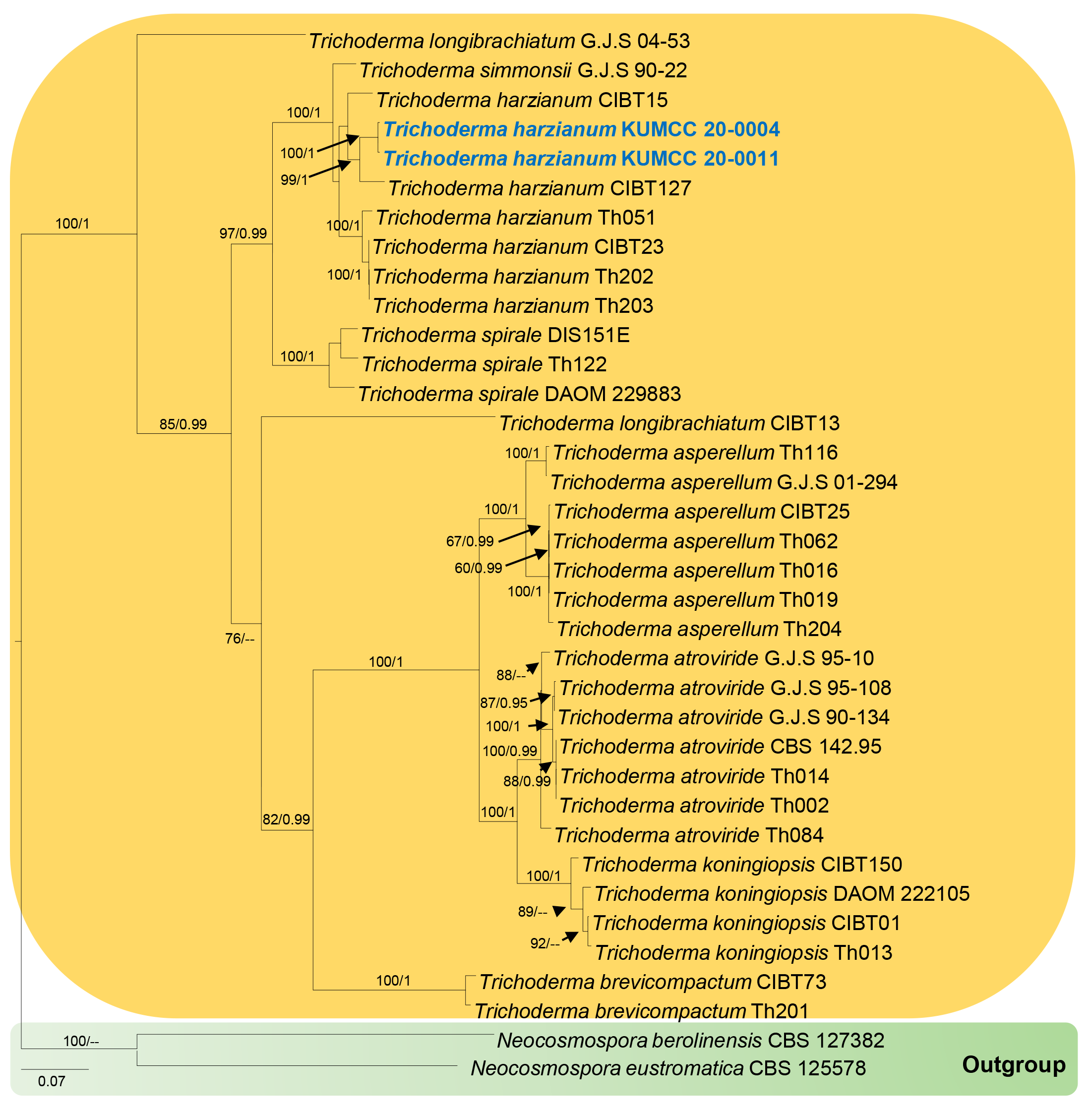


**Supplementary figure 9.** **Phylogram generated from RAxML analysis based on combined RPB2 and TEF1 sequence data.** There were 36 strains included in the combined sequence analysis, which comprise 1814 characters with gaps. *Neocosmospora berolinensis* (CBS 127382) and *Neocosmospora eustromatica* (CBS 125578) were used as the outgroup taxon. Tree topology of the ML analysis was similar to the BYPP. The best scoring RAxML tree with a final likelihood value of -11330.045995 is presented. The matrix had 949 distinct alignment patterns, with 36.38% undetermined characters or gaps. Estimated base frequencies were as follows: A = 0.235235, C = 0.268180, G = 0.227936, T = 0.268650; substitution rates AC = 1.359890, AG = 3.739938, AT = 1.124462, CG = 0.891245, CT = 6.007058, GT = 1.000000; gamma distribution shape parameter a = 0.388131. Bootstrap support values for ML equal to or greater than 60% and BYPP from MCMC analyses equal to or greater than 0.95 are given above/below the nodes. The ex-type strains are indicated in bold. Newly generated sequences are indicated in blue.


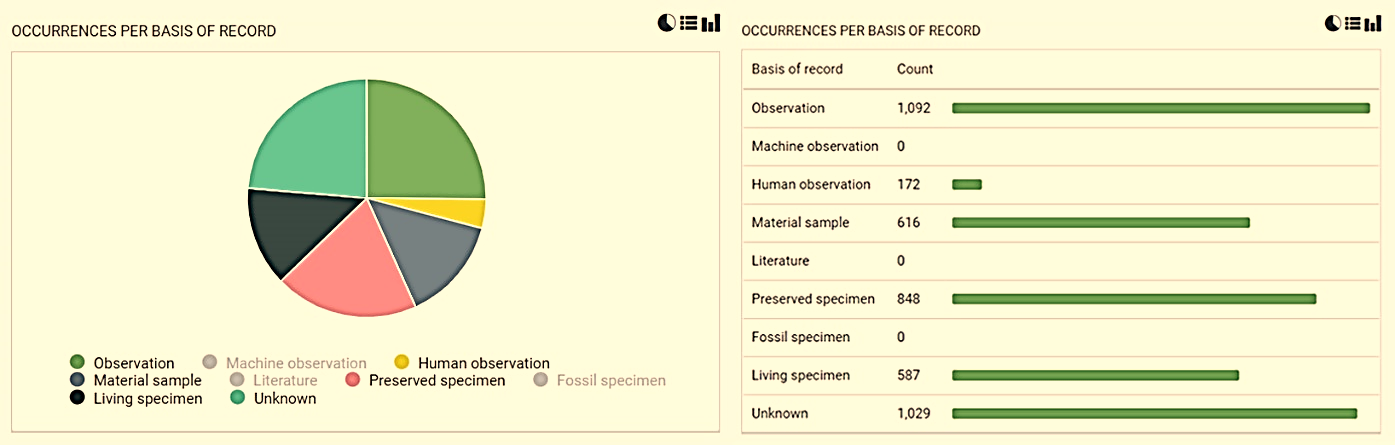


**Supplementary figure 10. *Trichoderma harzianum* occurrences during 1900-2017 with 2,465 records were obtained from Global Biodiversity Information Facility (accessed on 7 March 2020).**
