# Supplementary material for: Discovery of novel fungal species and pathogens on bat carcasses in a cave in Yunnan Province, China
Source: Emerg Microbes Infect. 2020 Jul 9;9(1):1554–66. doi: 10.1080/22221751.2020.1785333 (PMC7473127; doi:10.1080/22221751.2020.1785333)
Supplement: Supplemental Material [file TEMI_A_1785333_SM2640.zip › Supplementary files/Supplementary_tables.doc]

**Table 1.** Species of fungi on bat obtained in this study.

| **No.** | **Species name** | **Original code** | **Culture collection number** |
| --- | --- | --- | --- |
| 1 | *Fusarium incarnatum* | BatC | KUMCC 20-0006 |
| 2 | *Mortierella multispora* | BatB | KUMCC 20-0005 |
| 3 | *Mortierellarhinolophicola* | BatE | KUMCC 20-0008 |
|  |  | BatK | KUMCC 20-0014 |
| 4 | *Mortierella yunnanensis* | BatF | KUMCC 20-0009 |
|  |  | BatJ | KUMCC 20-0013 |
| 5 | *Mucor hiemalis* | BatG | KUMCC 20-0010 |
| 6 | *Neocosmospora pallidimors* | BatD | KUMCC 20-0007 |
|  |  | BatI | KUMCC 20-0012 |
| 7 | *Trichoderma harzianum* | BatA | KUMCC 20-0004 |
|  |  | BatH | KUMCC 20-0011 |

**Table 2.Gene regions and primers used in this study.**

| **Genes** | **Primers (forward/reverse)** | **PCR condition references** | **Primers references** |
| --- | --- | --- | --- |
| ***Fusarium*** | | | |
| TUB2 | T1/T2 | Herron et al. [1], Santos et al. [2] | O’Donnell &Cigelnik [3] |
| TEF1 | EF1/EF2 |  | O’Donnell et al. [4] |
| ***Mortierella*** | | | |
| ITS | ITS5/ITS4 | Wagner et al. [5] | White et al. [6] |
| LSU | LR0R/LR5 |  | Vilgalys& Hester [7], Vilgalys& Sun [8] |
| ***Mucor*** | | | |
| ITS | ITS5/ITS4 | Madden et al. [9] | White et al. [6] |
| ***Neocosmospora*** | | | |
| ITS | ITS4/ITS5 | Sandoval-Denis et al. [10, 11] | White et al. [6] |
| LSU | LR0R/LR5 |  | Vilgalys& Hester [7], Vilgalys& Sun [8] |
| RPB2 | 5f/7cr or 7cf/11ar |  | Liu et al. [12] |
| TEF1 | EF-1/EF-2 |  | O’Donnell et al. [4] |
| ***Trichoderma*** | | | |
| TEF1 | EF1-728F/TEF1 | Smith et al. [13], Nuankaew et al. [14] | Carbone & Kohn [15] |
| RPB2 | 5f/7cr |  | Liu et al. [12] |

**References**

1. Herron DA, Wingfield MJ, Wingfield BD, Rodas CA, Marincowitz S, Steenkamp ET. Novel taxa in the *Fusarium fujikuroi* species complex from *Pinus* spp. Studies in Mycology. 2015;80:131–150.

2. Santos AC, Trindade JV, Lima CS, Barbosa RD, da Costa AF, Tiago PV, de Oliveira NT. Morphology, phylogeny, and sexual stage of *Fusarium caatingaense* and *Fusarium pernambucanum*, new species of the *Fusarium incarnatum-equiseti* species complex associated with insects in Brazil. Mycologia. 2019;111(2):244–259.

3. O'Donnell K, Cigelnik E. Two divergent intragenomic rDNA ITS2 types within a monophyletic lineage of the fungus fusariumare nonorthologous. Molecular phylogenetics and evolution. 1997;7(1):103–116.

4. O’Donnell K, Kistler HC, Cigelnik E, Ploetz RC. Multiple evolutionary origins of the fungus causing Panama disease of banana: concordant evidence from nuclear and mitochondrial gene genealogies. Proceedings of the National Academy of Sciences. 1998;95(5):2044–2049.

5. Wagner L, Stielow JB, de Hoog GS, Bensch K, Schwartze VU, Voigt K, Alastruey-Izquierdo A, Kurzai O, Walther G. A new species concept for the clinically relevant *Mucor circinelloides* complex. Persoonia-Molecular Phylogeny and Evolution of Fungi. 2019.

6. White TJ, Bruns T, Lee SJ, Taylor J. Amplification and direct sequencing of fungal ribosomal RNA genes for phylogenetics. PCR protocols: a guide to methods and applications. 1990;18(1):315–322.

7. Vilgalys R, Hester M. Rapid genetic identification and mapping of enzymatically amplified ribosomal DNA from several Cryptococcus species. Journal of Bacteriology. 1990;172(8):4238–4246.

8. Vilgalys R, Sun BL. Ancient and recent patterns of geographic speciation in the oyster mushroom Pleurotus revealed by phylogenetic analysis of ribosomal DNA sequences. Proceedings of the National Academy of Sciences. 1994;91(10):4599–4603.

9. Madden AA, Stchigel AM, Guarro J, Sutton D, Starks PT. *Mucor nidicola* sp. nov., a fungal species isolated from an invasive paper wasp nest. International journal of systematic and evolutionary microbiology. 2012;62(7):1710–1714.

10. Sandoval-Denis M, Crous PW. Removing chaos from confusion: assigning names to common human and animal pathogens in *Neocosmospora*. Persoonia: Molecular Phylogeny and Evolution of Fungi. 2018;41:109.

11. Sandoval-Denis M, Lombard L, Crous PW. Back to the roots: a reappraisal of *Neocosmospora*. Persoonia-Molecular Phylogeny and Evolution of Fungi. 2019.

12. Liu YJ, Whelen S, Hall BD. Phylogenetic relationships among ascomycetes: evidence from an RNA polymerse II subunit. Molecular biology and evolution. 1999;16(12):1799–1808.

13. Smith A, Beltrán CA, Kusunoki M, Cotes AM, Motohashi K, Kondo T, Deguchi M. Diversity of soil-dwelling *Trichoderma* in Colombia and their potential as biocontrol agents against the phytopathogenic fungus Sclerotinia sclerotiorum (Lib.) de Bary. Journal of general plant pathology. 2013;79(1):74–85.

14. Nuankaew K, Sotome K, Lumyong S, Boonlue S. *Trichoderma polyalthiae* sp. nov., an endophytic fungus from Polyalthia debilis. Phytotaxa. 2018;371(5):273.

15. Carbone I, Kohn LM. A method for designing primer sets for speciation studies in filamentous ascomycetes. Mycologia. 1999;91(3):553–556.
